# Deciphering Dimerization Modes of PAS Domains: Computational and Experimental Analyses of the AhR:ARNT Complex Reveal New Insights Into the Mechanisms of AhR Transformation

**DOI:** 10.1371/journal.pcbi.1004981

**Published:** 2016-06-13

**Authors:** Dario Corrada, Anatoly A. Soshilov, Michael S. Denison, Laura Bonati

**Affiliations:** 1 Department of Earth and Environmental Sciences, University of Milano-Bicocca, Milan, Italy; 2 Department of Environmental Toxicology, University of California, Davis, Davis, California, United States of America; Koç University, TURKEY

## Abstract

The Aryl hydrocarbon Receptor (AhR) is a transcription factor that mediates the biochemical response to xenobiotics and the toxic effects of a number of environmental contaminants, including dioxins. Recently, endogenous regulatory roles for the AhR in normal physiology and development have also been reported, thus extending the interest in understanding its molecular mechanisms of activation. Since dimerization with the AhR Nuclear Translocator (ARNT) protein, occurring through the Helix-Loop-Helix (HLH) and PER-ARNT-SIM (PAS) domains, is needed to convert the AhR into its transcriptionally active form, deciphering the AhR:ARNT dimerization mode would provide insights into the mechanisms of AhR transformation. Here we present homology models of the murine AhR:ARNT PAS domain dimer developed using recently available X-ray structures of other bHLH-PAS protein dimers. Due to the different reciprocal orientation and interaction surfaces in the different template dimers, two alternative models were developed for both the PAS-A and PAS-B dimers and they were characterized by combining a number of computational evaluations. Both well-established hot spot prediction methods and new approaches to analyze individual residue and residue-pairwise contributions to the MM-GBSA binding free energies were adopted to predict residues critical for dimer stabilization. On this basis, a mutagenesis strategy for both the murine AhR and ARNT proteins was designed and ligand-dependent DNA binding ability of the AhR:ARNT heterodimer mutants was evaluated. While functional analysis disfavored the HIF2α:ARNT heterodimer-based PAS-B model, most mutants derived from the CLOCK:BMAL1-based AhR:ARNT dimer models of both the PAS-A and the PAS-B dramatically decreased the levels of DNA binding, suggesting this latter model as the most suitable for describing AhR:ARNT dimerization. These novel results open new research directions focused at elucidating basic molecular mechanisms underlying the functional activity of the AhR.

## Author Summary

Computational modeling combined with experimental validation may give insight into structural and functional properties of protein systems. The basic Helix-Loop-Helix PER-ARNT-SIM (bHLH-PAS) proteins show conserved functional domains despite the broad range of functions exerted by the different systems. Within this protein family, the Aryl hydrocarbon Receptor (AhR) is known to mediate the toxic effects of a number of environmental contaminants, including dioxins and dioxin-like chemicals, and it also exerts other biochemical and physiological effects. Despite the absence of experimentally determined structures, theoretical models of the AhR PAS domains developed on the basis of homologous systems have allowed understanding of some aspects of the molecular mechanisms underlying its function. In this work we present alternative structural models of the transcriptionally active complex of AhR with the AhR Nuclear Translocator (ARNT) protein. Computational characterization of the modeled protein-protein interaction interfaces guided the design of mutagenesis experiments, and evaluation of the DNA binding ability of the resulting AhR:ARNT dimer mutants allowed validation of the models and selection of the most reliable one. These findings open new research directions for understanding the molecular mechanisms underlying the functional activity of the AhR.

## Introduction

The Aryl hydrocarbon Receptor (AhR) is a *basic* Helix-Loop-Helix PER-ARNT-SIM (bHLH-PAS)-containing transcription factor that responds to a variety of structurally diverse exogenous and endogenous chemicals with the modulation of gene expression and production of diverse biological and toxic effects in a wide range of species and tissues [1]. Early studies were mainly focused on the role of AhR in mediating the biochemical response to xenobiotics and the toxic effects of environmental contaminants such as halogenated aromatic hydrocarbons (including dioxins and dioxin-like compounds). Recent studies have revealed endogenous regulatory roles for the AhR in normal physiology and development, including the pivotal role that this ligand-dependent transcription factor plays in the differentiation and/or affinity maturation of several key immune cells important in both innate and acquired immune response [1]. These findings have generated a renewed interest in understanding the molecular mechanisms of activation and their contribution to different complex physiological roles of the receptor [1–3].

In the classical mechanism of action [1], the AhR is maintained in its inactive form in the cytosol as part of a larger protein complex containing hsp90, XAP2, and p23. Following binding of ligand (agonist) to the AhR, the AhR protein complex translocates into the nucleus and its dimerization with the AhR Nuclear Translocator (ARNT) protein results in the release of the AhR from its cytosolic protein partners and conversion into its high-affinity DNA binding form. Binding of the ligand:AhR:ARNT complex to its specific DNA recognition site, the Dioxin Responsive Element (DRE), stimulates transcription of adjacent genes and the production of the spectrum of biological and toxic effects of AhR ligands [1,3].

Extensive mutagenesis and deletion analysis has been carried out to define the functional domains of the AhR and ARNT proteins and these experimental studies have identified a number of domains including those for DNA binding (bHLH), AhR:ARNT dimerization (HLH, PAS-A and PAS-B), ligand binding (PAS-B), hsp90 binding (bHLH and PAS-B) and transcriptional activation/coactivator binding (glutamine rich region) [4–6]. From a functional point of view, ligand binding to the AhR not only leads to a conformation change that exposes its nuclear localization sequence, facilitating nuclear translocation, but also exposes the PAS-A dimerization domain [7]. Once in the nucleus, the binding of ARNT to the exposed PAS-A domain progressively displaces hsp90 and its associated proteins and leads to subsequent interactions of ARNT with the second dimerization site present in the AhR bHLH domain [5,7]. Dimerization of the AhR and ARNT occurs primarily through the HLH and PAS-A domains, and while these domains appear sufficient on their own to allow transformation of the AhR:ARNT complex into its high affinity DNA binding form, deletion mutagenesis and DNA binding analysis have revealed that the PAS-B domain appears to be critically involved in initiation of AhR:ARNT dimerization [4,6].

AhR dimerization is a key step in the AhR signaling pathway and increased understanding of the PAS domain dimerization modes would provide further insights into mechanisms of ligand-dependent transformation of the AhR into its transcriptionally active DNA binding form. Moreover, it may provide avenues in which to finally gain insights into the ligand-dependent differences in AhR functionality. Despite elucidation of the role of the bHLH and PAS domains in the AhR:ARNT dimerization, the absence of experimentally determined structures for these proteins has hampered detailed analysis and understanding of the dimer geometry as well as of the molecular determinants of its stability. Recently however, several experimental structures of dimeric forms of PAS domains in other bHLH-PAS proteins have been become available, providing insights into the interactions between these domains [8–13].

bHLH-PAS proteins show conserved N-terminal domains as well as similarities in the mechanisms of action, despite the broad range of functions exerted by these proteins in different systems [2,14]. Their N-terminal domains include a common motif for DNA-binding (i.e. basic amino acids) attached to a HLH protein dimerization domain, followed by the PAS-A and PAS-B domains. Both PAS domains contribute to dimerization between bHLH-PAS partners, but show distinct functions. PAS-A is primarily a dimerization domain, whereas PAS-B is mainly important for sensing environmental or physiological signals, in addition to providing a second dimerization interface. Class I bHLH-PAS proteins (including AhR, the hypoxia-inducible factor α (HIF2α), the circadian locomotor output cycles kaput (CLOCK), and some neuronal factors) have the functional role of binding small molecules (AhR) or sensing environmental stimuli (i.e., hypoxic conditions for HIF2α). These proteins do not dimerize with other Class I proteins. Class II bHLH-PAS proteins (ARNT, ARNT2, and brain muscle ARNT-like (BMAL)) have regulatory roles. They are capable of forming dimers with one or more class I proteins and the resulting heterodimers gain the ability to bind to specific DNA sites to regulate specific target genes [2,14].

**Fig 1:**
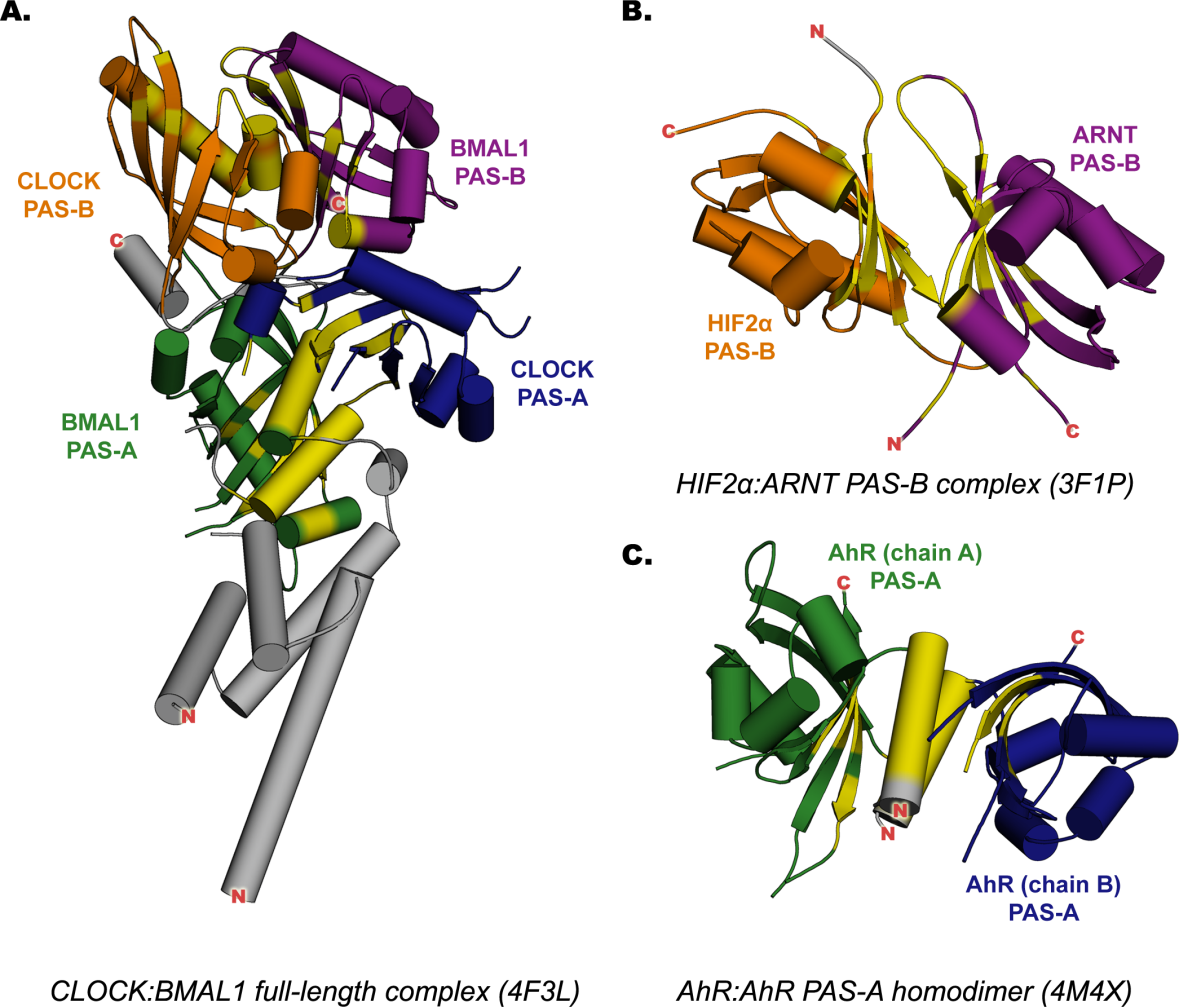
X-ray structures of the templates adopted for homology modeling. The PAS-A domains are colored in green and blue, the PAS-B domains in orange and violet; the residues involved in the dimerization interfaces are highlighted in yellow. (A) Murine CLOCK:BMAL1 complex, N-terminal region. (B) Human HIF2α:ARNT complex, PAS-B dimer. (C) Murine PAS-A AhR homodimer.

The structural and functional conservation of PAS proteins suggests that structural information derived from bHLH-PAS protein dimers can be exploited for modeling the AhR:ARNT PAS domain dimerization mode. On the other hand, comparison of the recently available crystal structures of these dimers [11–13] (Fig 1) shows that, although the PAS fold is well conserved, different orientations and interfaces appear to be involved in different PAS protein dimers.

The human HIF2α:ARNT PAS-B heterodimer, which was made amenable to crystallization by creating a highly stable HIF2α-R247E and ARNT-E362R double mutant [11], shows an antiparallel orientation of the two domains, with the β-sheet interfaces mediating protein-protein interaction (Fig 1B). In addition to the *apo* form, some structures of the same dimer containing artificial ligands demonstrated that the HIF2α internal cavity can bind ligands and that such ligands can modulate the HIF2α:ARNT interaction [8–11]. The murine CLOCK:BMAL1 heterodimer was the first crystal structure solved for a dimer consisting of intact bHLH-PASA-PASB domains [13]. It shows a roughly parallel orientation of the two proteins (Fig 1A). The PAS-B domains twist to align the interaction surfaces, consisting of the helical face of CLOCK and the β-sheet face of BMAL1. Each PAS-A mediates the heterodimeric interaction by a N-terminal extra-domain α-helix packed between the β-sheets surfaces. Finally, the murine AhR PAS-A homodimer [12], represents the first AhR derived protein structure, shows a dimerization interface similar to that of the CLOCK:BMAL1 PAS-A domains, including the extra-domain α-helices and a portion of the β-sheets surfaces, but with a slightly different reciprocal orientation of the two domains (Fig 1C).

An additional PAS dimer structure, the murine Period (PER) homodimer including both the PAS-A and the PAS-B domains has been reported [15,16]. However, PER lacks the bHLH motif, and the N-terminal portion of the PAS-A domains shows a disordered region instead of the α-helix present in the bHLH-PAS protein structures. The presence of this helix was shown to be critical for both the dimerization and the transcriptional activity of the AhR:ARNT complex [12]. Moreover, the unusual fold of the PER PAS-A domains may also influence the reciprocal orientation of the PAS-B domains, due to the short linker connecting the PAS domains and the presence of a secondary interaction interface of PAS-A:PAS-B type. For these reasons the X-ray structure of the PER homodimer appeared unsuitable for modeling the AhR:ARNT PAS complex.

In this contribution we aim to unveil the structural mode of dimerization of AhR:ARNT and identify the essential interacting interfaces. We describe the development and validation of a structural model of the murine AhR:ARNT PAS domain dimer using three X-ray structures of bHLH-PAS domain complexes described above [11–13] as structural templates for the homology modeling stages. The initial hypothesis is that AhR presents a PAS-B dimerization mode similar to that of HIF2α, given that the two proteins share the same dimerization partner, ARNT, and that several chemicals were observed to bind to the PAS-B of both proteins [17]. However, different reciprocal orientation of both the PAS-A and the PAS-B domains, described by the structural templates available for bHLH-PAS proteins, were examined and two alternative dimerization modes were considered for each domain. With the aim of predicting the most reliable dimerization mode, a number of geometrical and energetic computational evaluations were combined to analyze in depth the physico-chemical characteristics of each model at the dimerization interface and to predict the residues that mostly contribute to the dimer stabilization. Following the computational prediction, a set of mutagenesis experiments of both the murine AhR and ARNT proteins was designed and ligand-dependent DNA binding ability of the AhR:ARNT heterodimer mutants was utilized as a measure of normal functional AhR:ARNT dimerization in order to select the most reliable structural model for the AhR:ARNT PAS-A and PAS-B dimers.

## Results

### Template selection and modeling strategies

Homology modeling of the murine AhR:ARNT PAS domain dimers was performed adopting the following X-ray structures of mammalian PAS domain dimers: the human HIF2α:ARNT PAS-B heterodimer (PDB 3F1P) [11]; the murine CLOCK:BMAL1 heterodimer, inclusive of the bHLH, PAS-A and PAS-B domains (PDB 4F3L) [13]; and the murine AhR PAS-A homodimer (PDB 4M4X) [12] (Fig 1). In these structures, each individual domain contains both the secondary structure (SS) elements and the topological arrangement typical of PAS domains: a five-stranded anti-parallel β-sheet (with strands in the order 2-1-5-4-3), a bundle of three short helices, and a long α-helix, known as the helical connector (see S1 Fig). The PAS-A and PAS-B domain folds mainly differ in the length of connecting loops, while the PAS-A domains contain an additional N-terminal α-helix (A’).

Given that the template X-ray structures show slightly different reciprocal orientation of the PAS-A domains (S2A Fig) and completely different PAS-B dimerization interfaces (S2B Fig), to analyze all the putative dimerization modes of these domains in the AhR:ARNT complex, a two step modeling strategy was devised. In the first step, modeling of the individual PAS-A and PAS-B structures of the mouse AhR and ARNT (mAhR and mARNT) was performed starting from the template structure with the highest sequence identity available for each domain. In the second step, different models of the PAS-A and PAS-B dimers were assembled from the protomer models, according to the different dimerization modes described by the template structures. This approach allows detailed examination of the protomer structural rearrangements in the dimerization process and facilitates direct comparison of the different dimer models.

The mAhR PAS-A domain was extracted from the crystallographic structure of the mAhR PAS-A homodimer (PDB 4M4X), while for the mARNT PAS-A domain a homology model was developed, using as template the structure of the mBMAL1 PAS-A domain in the CLOCK:BMAL1 dimer (PDB 4F3L; similarity: 75.2%, identity: 55.0%). Both experimental PAS-A structures lack several residues that map to extended loops connecting the SS elements of the domain core (S1 Fig), significantly distant from the PAS dimerization interface. Since *ab initio* loop refinement methods applied to such long disordered regions may be error prone [18], the missing regions were filled by grafting the atomic coordinates of the topologically equivalent PAS-B loops onto the PAS-A structures (as detailed in the Methods Section). The DOPE profiles confirm the quality of both models and the regions surrounding the gaps appear to be not perturbed by the insertion of the grafted loops (S3 Fig).

Among the structural models previously proposed and validated by our group for the mAhR PAS-B domain [17,19], the one obtained from multiple HIF2α PAS-B *holo* structures [17] (similarity: 52.3%, identity: 30.8%) was used as the mAhR PAS-B protomer model. Since ligand binding to the PAS-B stimulates the AhR nuclear translocation and its subsequent dimerization with ARNT, the *holo* form of the domain obtained by docking the high affinity ligand 2,3,7,8-tetrachlorodibenzo-p-dioxin (TCDD) [17] was adopted. The ARNT PAS-B domain was derived from the X-ray structure of the human isoform (HIF2α:ARNT PAS-B heterodimer, PDB 3F1P; similarity: 98.2%, identity: 96.4%) by applying a PyMOL tool for *in silico* mutagenesis [20] at the following positions: V357I, R362E, N433T and Q434R.

In the second modeling step, to obtain the complete structure of the AhR:ARNT PAS-A dimer two alternative models, each derived from one of the templates (CLOCK:BMAL1 and AhR:AhR), were developed by introducing a further refinement of the spatial atomic coordinates in the protomer models, mainly involving the A' α-helices. For the mAhR:ARNT PAS-B dimer two alternative models were built, starting from the protomer models and by using the two distinctly different template structures of the HIF2α:ARNT and CLOCK:BMAL1 complexes. Since the helical bundle region of the CLOCK PAS-B domain is a structurally disordered region rich in prolines, no further refinement based on the CLOCK structure was conducted in this region (residues: 295–314 in mAhR).

**Fig 2:**
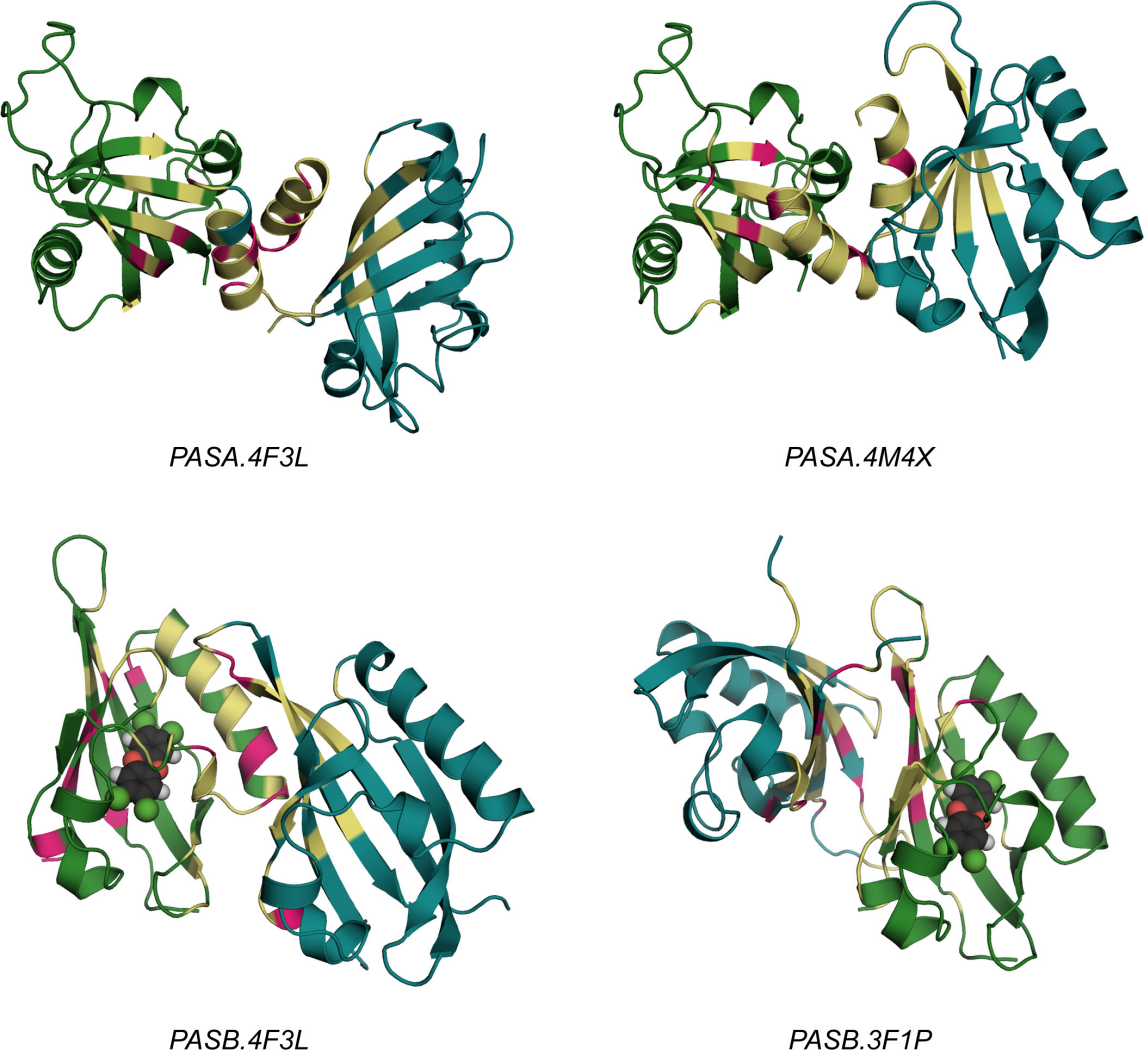
The four PAS dimer models proposed. The AhR domains are colored in green, the ARNT ones in cyan. Residues defining the protein-protein interface (according to PISA analysis) are highlighted in yellow and residues defining the specific ∆*G signatures* (according to the Rank Products approach) are highlighted in magenta. For the PAS-B dimers, the AhR bound ligand TCDD is depicted in spheres.

The two alternative models developed for the AhR:ARNT PAS-A dimer and the two for the PAS-B dimer (Fig 2) were termed *PASA*.*4F3L*, *PASA*.*4M4X*, *PASB*.*4F3L* and *PASB*.*3F1P*, respectively, consistent with the PDB ID of the structural template.

Sequence alignments used to obtain the dimer models are presented in S4 Fig. Sequence identities with the templates (S1 Table) are above the “twilight zone” threshold (30–40% identity) that was proposed to infer similarity in the interactions of protein-protein complexes [21] and identity and similarity further increase at the dimerization interfaces (S1 Table). The SS elements predicted for the targets are highly consistent with those of the templates (see the RMSD values in S2 Table). The stereochemical quality, assessed by PROCHECK [22], is good, with 87–95% of residues found in the most favored areas of the Ramachandran plot, and no residues in the disallowed regions. The overall G-factors range from -0.18 to 0.15 (S2 Table). The overall Z-scores calculated with the ProSA [23,24] validation method range from -4.03 to -8.03, i.e. within experimentally determined values for protein chains in the current PDB (S2 Table and S5 Fig).

### Characterization of the dimerization interfaces

The geometrical and energetic features of the AhR:ARNT PPI interfaces for either the PAS-A and PAS-B dimers were compared between the two alternative models in each case, in order to identify the model with the most reliable dimerization mode.

The number of residues involved in the dimerization interface of each protomer model obtained by PISA analysis [25], the characteristics of their intermolecular interactions derived by using LigPlot+ [26], and the changes in the total solvent accessible surface area upon complexation (ΔSASA, calculated using NACCESS [27]) are reported in Table 1. The same analyses were performed for the dimerization interfaces of the templates, to allow a direct comparison with the models (data in brackets in Table 1).

**Table 1:** Overall evaluation of the dimerization interfaces of the models and the templates adopted (in brackets).

	*PASA*.*4F3L*		*PASA*.*4M4X*		*PASB*.*4F3L*		*PASB*.*3F1P*	
	AhR (CLOCK)	ARNT (BMAL1)	AhR (AhR[A]^a^)	ARNT (AhR[B]^a^)	AhR (CLOCK)	ARNT (BMAL1)	AhR (HIF2a)	ARNT (ARNT)
**ΔSASA**^b^ **(Å**^**2**^**)**	809.7 (1159.4)	846.0 (1144.3)	778.3 (702.5)	809.7 (728.0)	526.3 (618.1)	629.0 (733.9)	902.1 (959,8)	796.8 (965.3)
**Residues**^c^	26 (30)	26 (30)	27 (25)	30 (23)	27 (27)	20 (19)	28 (30)	35 (34)
**Ele**^d^	8 (11)	4 (10)	2 (3)	2 (2)	3 (3)	3 (4)	4 (3)	4 (4)
**VdW**^d^	7 (11)	9 (12)	13 (11)	13 (8)	7 (11)	6 (9)	12 (14)	13 (16)

ΔSASA, variation in solvent- accessible surface area; Ele, non-bonded electrostatic interactions; VdW, non-bonded Van der Waals interactions

^a^: the letters in square brackets indicates the polypeptide chains of the crystallographic structure

^b^: calculated by means of NACCESS method

^c^: amount of residues belonging to the PPI interface predicted by PISA

^d^: non-bonded interactions are defined by means of LigPlot+ and PISA tools

The results in Table 1 reveal that the PAS-A dimer models show similar interfaces, in terms of area and total number of residues involved, suggesting that the AhR and the ARNT domains contribute equally to the interaction. The *PASA*.*4M4X* model is characterized by more van der Waals interactions, while that of *PASA*.*4F3L* shows more electrostatic interactions (H-bonds and salt bridges). In the PAS-B dimer models, the contributions provided by the AhR and ARNT domains are clearly different. In *PASB*.*4F3L*, where the helical bundle of AhR interacts with the β-sheet of ARNT, ARNT provides a more extended interface area, while in *PASB*.*3F1P*, characterized by the interaction between the β-sheets of the two domains, AhR contributes a larger interface. Moreover, the *PASB*.*3F1P* model exhibits more hydrophobic (van der Waals) interactions.

No substantial differences emerge between the surface extension and the number of interface residues of each model and those of the related template (this is also highlighted in the sequence alignment, S4 Fig). However, as detailed below, several differences emerge in the residue types and consequently in the intermolecular interactions with respect to the templates.

The characteristics of the inter-domain interactions were analyzed in depth using multiple approaches. A preliminary comparison between the per-residue contributions to the global binding free energy in the two alternative dimer models was performed by the Rank Products method [28,29], and applied to MM-GBSA multiple evaluations (for more details see Methods section). Next, the pattern of residues showing significantly different contributions to ∆*Gbinding* in a given model was termed its ∆*G signature* (*vide infra*). The MM-GBSA ∆*Gbinding* value of each model is related to overall dimer stability, and the residue pairwise contributions to this property, calculated using Energy Decomposition analysis [29,30] (see Methods section), were used to localize the most significant energetic couplings in the dimer structure. The Electrostatic Potential Surface, obtained by DelPhi [31], was used to estimate the complementarity of charged/neutral regions at the dimerization interface. Finally, the *hot spot* prediction performed by HotPoint [32], KFC2 [33] and Robetta [34] methods allowed selection of a group of residues that significantly contributed to the binding mode and to the dimer stability.

#### PAS-A dimer models

**Fig 3:**
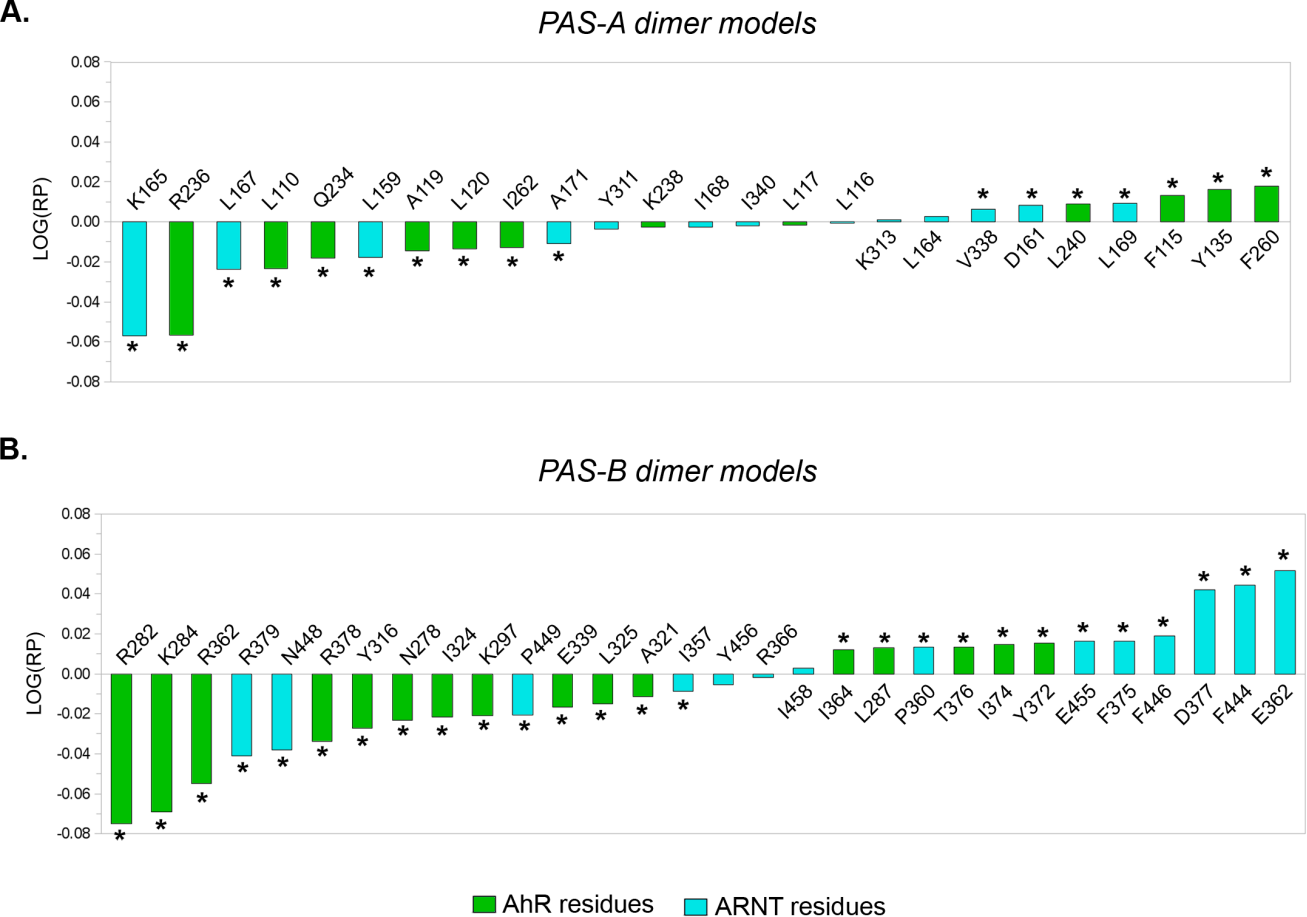
Rank Products profiles from the comparison of the PAS-A and PAS-B dimer models. Asterisks denotes residues with a significant LOG(*RP*) value (*e-value* ≤ 0.05 after 10,000 random permutations). (A) Residues with a negative LOG(*RP*) value define the *PASA*.*4F3L* ∆*G signature*; residues with a positive LOG(*RP*) value define the *PASA*.*4M4X* ∆*G signature*. (B) Residues with a negative LOG(*RP*) value define the *PASB*.*4F3L* ∆*G signature*; residues with a positive LOG(*RP*) value define the *PASB*.*3F1P* ∆*G signature*.

In the Rank Products profile shown in Fig 3A, residues with a negative LOG(*RP*) value define the ∆*G signature* of the *PASA*.*4F3L* model (i.e. they exert a relatively higher stabilizing effect in this model than in the *PASA*.*4M4X*). Conversely, residues with a positive LOG(*RP*) value define the ∆*G signature* of the *PASA*.*4M4X* model (see Methods for further details).

Almost all of the selected residues belong to both dimerization interfaces of the models, as predicted by PISA (S3 Table**)**, with the exception of AhR:L240 and ARNT:D161 (exclusive to the *PASA*.*4M4X* model). This confirms the similarity between the PAS-A dimer models. On the other hand, the distinct ∆*G signatures* highlight a somewhat different interaction pattern in each model. Unexpectedly, the 3D mapping of the ∆*G signatures* (Fig 2) shows how the residues that provide the most distinct contribution to ∆*Gbinding* are restricted to a sub-region of the dimerization interface and that the interactions provided from the AhR β-sheet are more significant than those from the ARNT β-sheet, for both the PAS-A dimer models.

**Fig 4:**
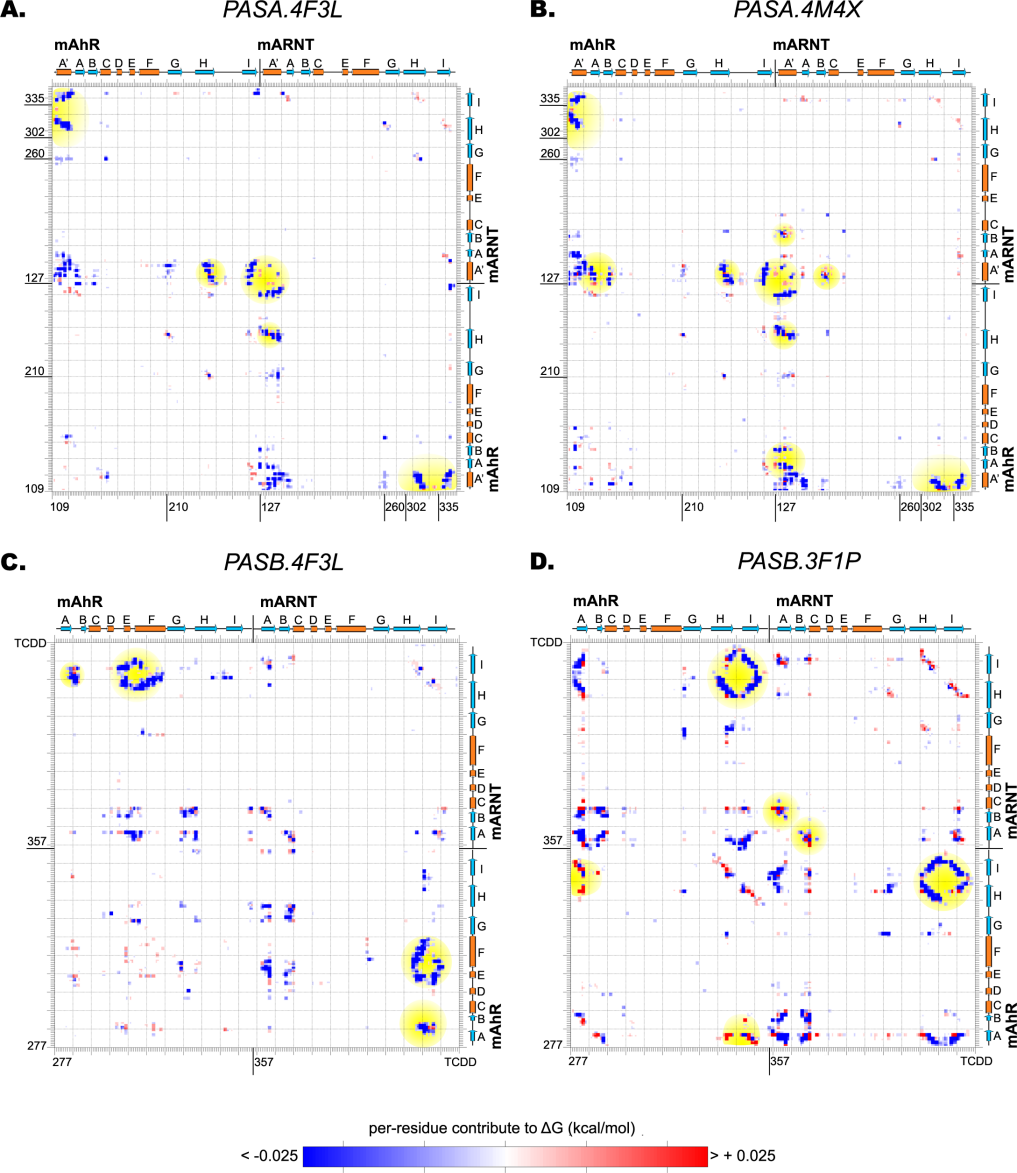
*Interaction energy matrices* of the PAS-A and PAS-B dimer models. The blue and the red spots identify the energy couplings that define stabilizing or destabilizing contributions, respectively, accounting for the overall Δ*Gbinding* of the complex. The spots discussed in the text are shaded in yellow. (A) *PASA*.*4F3L* dimer model. (B) *PASA*.*4M4X* dimer model. (C) *PASB*.*4F3L* dimer model. (D) *PASB*.*3F1P* dimer model.

The *PASA*.*4F3L* dimer model is characterized by a low value of Δ*Gbinding* (-40.07 kcal mol^-1^), indicating a good stability of the dimer. The decomposition of Δ*Gbinding* highlights stabilizing energetic intermolecular couplings between the H and I strands and the A' helix of the opposite domains (*interaction energy matrix*, Fig 4A).

**Fig 5:**
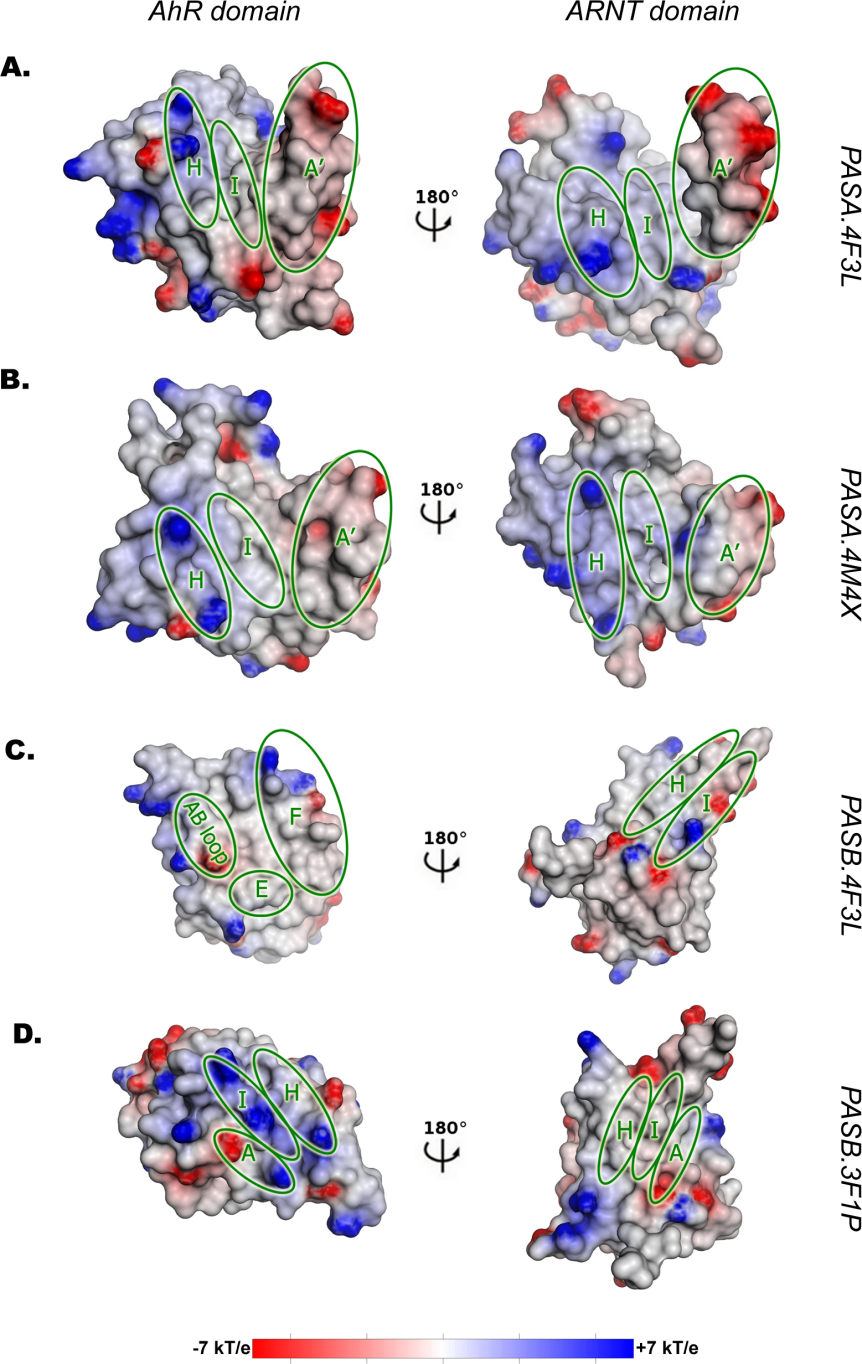
Electrostatic Potential Surface (EPS) of the dimer models. In each panel, the ARNT domain is rotated of 180 degree with respect to the AhR domain, to obtain a representation of the dimer as an “open book”. The potential range is defined in kT/e units according to the DelPhi software. The secondary structure elements mainly involved in the PPI definition are highlighted with green circles. (A) *PASA*.*4F3L* dimer model. (B) *PASA*.*4M4X* dimer model. (C) *PASB*.*4F3L* dimer model. (D) *PASB*.*3F1P* dimer model.

The electrostatic potential surfaces (EPS) of both the AhR and the ARNT PAS-A domains (Fig 5A) show a neutral cleft (hydrophobic region) in the central part of the dimerization interface (mainly defined by the I strand, the internal face of the A' helix and the N-terminal portion of the H strand), thus indicating good complementarity between the two domains. The more solvent exposed portion of the interface is characterized by regions in which the positive potential provided by residues in the C-terminal portion of the AhR H strand face the negative potential provided by residues in the A' helix of ARNT and vice-versa. The general EPS features of this model at the PPI interface resemble those of the CLOCK:BMAL1 template (S6A Fig), with some peculiarities in the more external regions of the interface of the AhR where fewer charged residues than in the CLOCK template are present.

**Fig 6:**
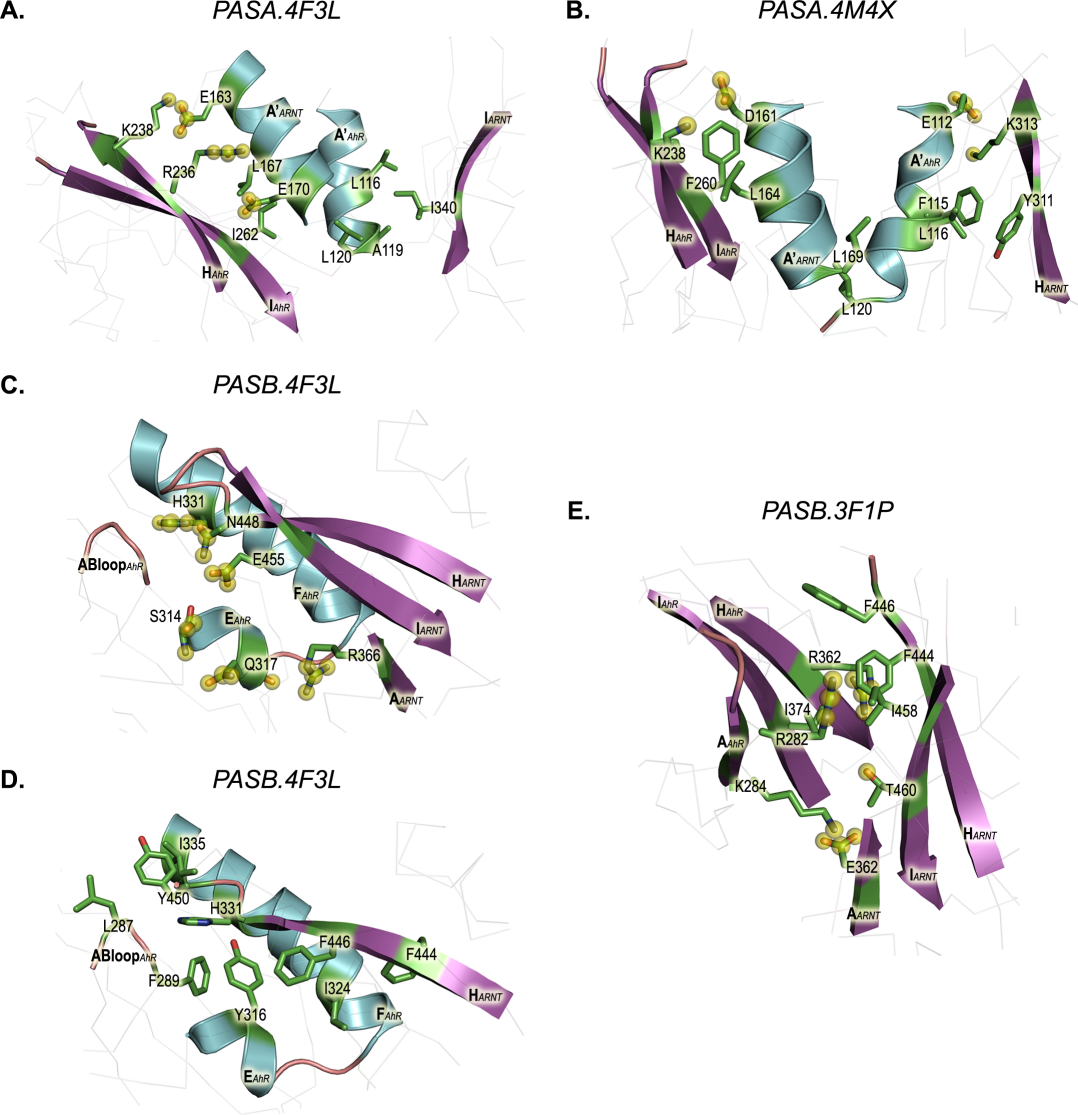
Residue contacts for the dimer models derived from the PPI prediction methods and visual inspection. Residues involved in electrostatic non-bonded interactions (i.e. H-bonds and/or salt bridges) are highlighted with yellow spheres. In both the AhR and ARNT domains, helices are shown in cyan, strands in purple. (A) *PASA*.*4F3L* dimer model. (B) *PASA*.*4M4X* dimer model. (C) and (D) PASB.4F3L dimer model. (E) PASB.3F1P dimer model.

The *hot spot* list collected by the different prediction methods confirms the interface energetic characteristics of the dimer model. The entire A' helix of ARNT is involved in the stabilization of the dimer but only the more buried portion of the A' helix of the AhR seems to be relevant (Fig 6A). The A' helix of ARNT contacts the H strand of AhR through only two electrostatic interactions (AhR:K238 to ARNT:E163 and AhR:R236 to ARNT:E170) and behind them an extended hydrophobic network of mainly aliphatic residues connects the A' helices and the I strands of the two domains. This includes the residues AhR:L116, AhR:A119, AhR:L120, AhR:I262, ARNT:L167, ARNT:I340, and most of them seem to be important for the stabilization of the dimer, according to the ranks proposed by all the *hot spot* prediction methods (Table 2).

**Table 2:** *Hot spot* list from PAS-A dimer models, with related scores from the PPI prediction tools herein adopted.

*PASA*.*4F3L*				*PASA*.*4M4X*			
Residue	Robetta	HotPoint	KFC2-A	Residue	Robetta	HotPoint	KFC2-A
**AhR:L116**	1.26	26.53	0.73	AhR:L110	0.51	32.94	-0.34
AhR:L117	0.68	39.46	0.01	AhR:E112	0.72	9.65	0.19
AhR:A119	0.00	12.33	0.10	AhR:F115	2.53	17.12	-0.30
**AhR:L120**	1.97	39.34	1.35	**AhR:L116**	2.41	28.67	0.71
AhR:R236	0.38	16.35	0.14	AhR:L117	0.44	41.45	0.80
AhR:F260	0.48	25.43	0.03	**AhR:L120**	1.40	33.14	-0.01
**AhR:I262**	0.95	43.23	1.13	AhR:V124	0.53	38.39	0.17
ARNT:L159	0.81	37.38	-1.12	AhR:L240	*na*	28.51	-2.25
ARNT:E163	-0.45	11.28	0.74	**AhR:F260**	2.21	27.04	1.20
ARNT:L164	0.52	24.70	-0.51	AhR:I262	0.54	25.90	-1.48
**ARNT:L167**	1.89	38.70	1.13	ARNT:D161	-0.05	13.08	0.54
ARNT:I168	0.81	39.90	0.54	**ARNT:L164**	0.85	16.13	0.38
ARNT:A171	0.00	25.54	-0.07	ARNT:I168	2.18	23.52	0.81
ARNT:L176	0.26	29.65	-1.90	**ARNT:L169**	0.73	38.99	-0.01
ARNT:Y311	2.33	14.40	-0.34	ARNT:L176	0.48	48.69	-0.99
**ARNT:I340**	0.98	30.43	0.42	ARNT:V338	0.54	28.24	-0.44
				ARNT:A339	*na*	22.49	-1.02
				ARNT:I340	0.92	25.74	-0.80

*na*: residue not predicted as hot spot for the specific method

Residues highlighted in bold mostly contribute to the dimer stabilization according to the scores proposed by all methods

The *PASA*.*4M4X* dimer model shows lower stability (Δ*Gbinding* = -27.77 kcal mol^-1^) than the *PASA*.*4F3L*. Nevertheless, the EPSs show similar properties (Fig 5B): the PAS-A domains of AhR and ARNT share a core characterized by a neutral potential surrounded by matched complementary areas of positive/negative potentials located on the most solvent exposed portions of the two domains. This model maintains the electrostatic complementarity observed in the AhR homodimer (S6B Fig), and the only differences from the template appear at the boundary of the interface due to a diverse distribution of the charged residues in ARNT.

The *interaction energy matrix* (Fig 4B) reveals how the energetic couplings that define the overall binding free energy involve more secondary structural elements with respect to the *PASA*.*4F3L* model. The A' helices of both the domains are in contact with the A and B strands (in addition to the H and I strands), showing partially destabilizing interactions. Moreover, the A' helix of ARNT is positioned in such a manner that a lysine (ARNT:K133) is oriented inward the hydrophobic core of the AhR interface. These two observations may explain the difference in binding free energy between the two PAS-A dimer models.

Unlike the *PASA*.*4F3L* model, the predicted *hot spots* for the *PASA*.*4M4X* span over the entire interface (Fig 6B). On both the N-terminal portions of the A' helices, acidic residues (AhR:E112 and ARNT:D161) appear involved in salt bridges formed with lysines located on the opposite H strands (ARNT:K313 and AhR:K238). Just beneath, two phenylalanines are highlighted by the prediction methods (AhR:F115 and AhR:F260). AhR:F115 appears to provide a stacking interaction with the aromatic ring of ARNT:Y311. These residues define the boundaries of the hydrophobic core of the dimerization interface, connecting the lower portion of the A' helices with the H and I strands. Among the aliphatic residues that populate this region, AhR:L116, AhR:L120, ARNT:L164, ARNT:L169 seem to be the most important for the stabilization of the dimer, according to the high scores generated by all prediction methods (Table 2).

The *hot spots* predicted for the PAS-A dimer models and those for the related templates only partially overlap (S4 Table). Several *hot spots* of the templates are not predicted for the models and vice-versa, even if most of them are conserved residues. Furthermore, the scores attributed by the prediction methods to the *hot spot* residues at conserved positions are often different. For example, AhR:L116 of the *PASA*.*4M4X* model is predicted as much more important than the same residue in the AhR homodimer template.

#### PAS-B dimer models

From the Rank Products profile shown in Fig 3B the residues with a negative LOG(*RP*) value define the ∆*G signature* of the *PASB*.*4F3L* model, while the residues with a positive LOG(*RP*) value defines the ∆*G signature* of the *PASB*.*3F1P*.

Several residues belong to only one of the two dimerization interfaces (S5 Table), thus confirming that the binding modes described for the two proposed PAS-B dimers are significantly different. Furthermore, the ∆*G signature* of the *PASB*.*4F3L* model includes numerous residues from the AhR domain, while that of the *PASB*.*3F1P* appears more balanced between the AhR and ARNT domains. Moreover, in the ∆*G signature* of the *PASB*.*4F3L* model various residues are exclusive to the dimerization interface of the *PASB*.*3F1P* (AhR: R282, K284, E339, R362, R378), as depicted in Fig 2. In agreement with the definition of ∆*G signature*, when we compare a query (*PASB*.*4F3L*) with a reference model (*PASB*.*3F1P*), the ∆*G signature* for the query model includes not only the most stabilizing residues for its dimerization interface, but also the most de-stabilizing ones for the reference interface. This identifies a number of interface residues that have a strong destabilizing effect in the *PASB*.*3F1P* model.

The *PASB*.*4F3L* dimer model, in which the β-sheet of the ARNT PAS-B domain faces the helical bundle of the AhR PAS-B, is characterized by a low value of Δ*Gbinding*, comparable to that of the PAS-A dimer models (-34.49 kcal mol^-1^). The EPSs reveal a hydrophobic groove on the AhR side (limited by the E, F helices and the AB loop,) where a complementary neutral region of ARNT (including the H and I strands and their connecting loops) can be accommodated (Fig 5C). The *interaction energy matrix* confirms strong stabilizing energetic couplings interconnecting these structural elements (Fig 4C). The EPSs at the dimerization interface are almost identical to those of the CLOCK:BMAL1 template, especially at the ARNT side (S7A Fig). In fact, despite this model shows the lowest sequence similarity with the template among the four models herein proposed (see S1 Table), it exhibits high similarity in the dimerization interface.

The *hot spots* list (Table 3) includes the hydrophobic network between the AhR groove and the ARNT H strand and highlights two distinct patches in the interface (Fig 6D). The lower portion hosts a bundle of aromatic residues, AhR:Y316, ARNT:F444 and ARNT:F446 (and also includes a non-hot spot residue, AhR:F289) with the AhR:I324 side chain inserted in the middle. In the upper patch, the aliphatic residues AhR:L287 and AhR:I335 define a narrow cleft into which tyrosine ARNT:Y450 is inserted. Residues AhR:Y316, AhR:I324, ARNT:F446 and ARNT:Y450 have the highest scores attributed by the prediction methods. Two *hot spot* polar residues AhR:H331 and ARNT:N448 were also found to be important for dimer stabilization. These residues, along with other polar and charged residues at the sides of the hydrophobic PPI interface, are involved in a stabilizing intermolecular network of electrostatic interactions (ARNT:R366 with AhR:Q317; ARNT:E455 with AhR:S314 (Fig 6C)).

**Table 3:** *Hot spot* list from PAS-B dimer models, with related scores from the PPI prediction tools herein adopted.

*PASB*.*4F3L*				*PASB*.*3F1P*			
Residue	Robetta	HotPoint	KFC2-A	Residue	Robetta	HotPoint	KFC2-A
AhR:L287	0.27	18.09	-1.74	AhR:I280	0.53	28.79	-0.62
**AhR:Y316**	2.46	32.79	0.86	AhR:R282	-0.56	32.56	1.47
**AhR:I324**	1.40	34.42	0.55	AhR:K284	-1.02	19.33	1.73
AhR:H331	1.99	30.18	-0.48	AhR:I292	0.24	27.17	-1.21
AhR:I335	0.27	25.67	-0.84	AhR:R362	-0.47	21.76	1.50
ARNT:F444	1.15	*na*	-1.28	AhR:I364	0.37	21.02	0.90
**ARNT:F446**	1.75	31.41	0.70	**AhR:I374**	1.12	33.85	0.71
ARNT:N448	0.80	17.14	0.00	AhR:T376	0.12	21.10	0.81
**ARNT:Y450**	1.60	20.23	0.40	ARNT:E362	0.21	25.51	0.25
ARNT:I458	0.57	26.09	-0.26	ARNT:I364	0.07	37.43	1.07
				ARNT:S442	-0.35	15.98	0.49
				**ARNT:F444**	0.63	30.42	0.63
				**ARNT:F446**	1.57	28.20	0.66
				ARNT:Y456	0.17	26.02	-0.55
				ARNT:I458	-0.67	33.85	1.40
				ARNT:T460	-0.06	25.72	1.43

*na*: residue not predicted as hot spot for the specific method

Residues highlighted in bold mostly contribute to the dimer stabilization according to the scores proposed by all methods

The dimerization interface of the *PASB*.*3F1P* model involves the β-sheets of both domains. The EPSs of the two domains show an unfavorable match in central region, with positive potential on the AhR surface facing a mainly neutral surface on ARNT (Fig 5D). The AhR region of positive potential is defined by three basic residues very close to each other: AhR:R282, AhR:K284 and AhR:R362. The sub-optimal interaction between the two domains, already suggested by the Rank Products profile (Fig 3B), is further confirmed by the positive value of the total binding free energy (Δ*Gbinding* = 1.73 kcal mol^-1^) that indicates an unstable dimer. Also, the *interaction energy matrix* reveals diffused destabilizing energy couplings between strands A, H and I of both domains (Fig 4D). In contrast, the EPSs of HIF2α and ARNT in the template show high electrostatic complementarity (S7B Fig). In this case, in spite of the high sequence similarity between the model and the template at the interface (S1 Table and S4 Fig), the few non-conserved charged residues in the AhR interface cause a significant reduction of the electrostatic complementarity and of the overall dimer stability with respect to the template.

Nevertheless, the three basic residues mentioned above were predicted as *hot spots* (Table 3). A visual inspection of the 3D structure of the model suggests some electrostatic interactions in which they may also be involved (e.g., a putative salt bridge between AhR:K284 and ARNT:E362 (Fig 6E)). However, these interactions appear buried and isolated by an ensemble of surrounding hydrophobic residues. In this context, the most important interacting residues possessing high scores in all *hot spot* prediction methods are AhR:I374, ARNT:F444 and ARNT:F446.

As previously observed for the PAS-A dimers, the PAS-B dimers demonstrate several differences between the *hot spot* list predicted for the templates and that for the models (S6 Table). Furthermore, in the *PASB*.*3F1P* model, several *hot spot* residues in positions topologically equivalent to the template have very different physico-chemical properties (see also S4 Fig); as previously evidenced by the comparison of EPSs (S7 Fig), this causes important differences in the interface complementarity

### Mutagenesis strategy

To validate the proposed PAS-A and PAS-B dimer models in order to establish the most reliable AhR:ARNT dimerization mode, a panel of point mutations was defined. The list of the proposed mutants is depicted in Table 4 and involves 11 residues in the AhR protein (6 in PAS-A and 5 in PAS-B) and 16 residues in the ARNT protein (7 in PAS-A and 9 in PAS-B).

**Table 4:** List of proposed AhR and ARNT dimerization mutations.

Model^a^	Protein	Domain	Region	Mutation
*PASA*.*4F3L*	AhR	PAS-A	helix A'	**A119R**^b^
*PASA*.*4F3L*	AhR	PAS-A	strand H	Q234R
*PASA*.*4F3L*	AhR	PAS-A	strand H	R236A
*PASA*.*4F3L*	ARNT	PAS-A	helix A'	E163A^c^
*PASA*.*4F3L*	ARNT	PAS-A	helix A'	**L167E**
*PASA*.*4F3L*	ARNT	PAS-A	strand I	I340R^b^^,^^d^
*PASA*.*4F3L*	ARNT	PAS-A	strand I	R342A
*PASA*.*4M4X*	AhR	PAS-A	helix A'	F115S
*PASA*.*4M4X*	AhR	PAS-A	strand H	K238A
*PASA*.*4M4X*	AhR	PAS-A	strand I	**F260S**^b^
*PASA*.*4M4X*	ARNT	PAS-A	helix A'	D161A^b^^,^^c^
*PASA*.*4M4X*	ARNT	PAS-A	helix A'	L169R
*PASA*.*4M4X*	ARNT	PAS-A	strand H	Y311Q
*PASB*.*4F3L*	AhR	PAS-B	loop AB	F289L^d^^,^^e^
*PASB*.*4F3L*	AhR	PAS-B	helix E	**Y316R**^d^
*PASB*.*4F3L*	AhR	PAS-B	helix F	**I324R**
*PASB*.*4F3L*	ARNT	PAS-B	strand A	R366A
*PASB*.*4F3L*	ARNT	PAS-B	strand H	F444E
*PASB*.*4F3L*	ARNT	PAS-B	strand H	**F446S**
*PASB*.*4F3L*	ARNT	PAS-B	strand H	**N448A**
*PASB*.*4F3L*	ARNT	PAS-B	loop HI	Y450Q^e^
*PASB*.*4F3L*	ARNT	PAS-B	strand I	**E455A**
*PASB*.*3F1P*	AhR	PAS-B	strand A	**K284D**^f^
*PASB*.*3F1P*	AhR	PAS-B	strand I	**I374W**^f^
*PASB*.*3F1P*	ARNT	PAS-B	strand A	E362K^f^
*PASB*.*3F1P*	ARNT	PAS-B	strand H	F444R
*PASB*.*3F1P*	ARNT	PAS-B	strand I	**I458W**^e^

Residues highlighted in bold are mutation in which an effective disrupting effect is observed and will be commented in the Discussion section

^a^: model for which the mutation should have a disrupting effect, on the basis of PPI hot spot prediction

^b^: other mutations in this position were reported by Rastinejad and co-workers [12]

^c^: other mutations in this position were reported by Chapman-Smith and co-workers [35]

^d^: other mutations in this position were reported by Denison and co-workers [36]

^e^: other mutations on topological equivalent positions were reported on the CLOCK:BMAL1 complex by Zhang and co-workers [13]

^f^: other mutations on topological equivalent positions were reported on the HIF2α:ARNT complex by Gardner and co-workers [11,37]

The specific mutagenesis positions were chosen based on the energetic/structural information provided from the EPS surfaces, the *interaction energy matrices*, and the *hot spot* predictions. An accurate visual inspection of the 3D structures helped us to hypothesize which positions are of most importance for each dimer, and which show unique features between the proposed alternative dimer models. The latter characteristic was verified by using the rank products profiles: most of the proposed mutated positions in a specific dimer model belong to the Δ*G signature* of that model (Fig 3).

Since the two PAS-A dimer models share a somewhat similar dimerization interface, the positions selected from both models map mainly onto the A’ α-helices and the H and I strands of both the AhR and ARNT proteins (Table 4). Both PAS-B dimer models suggest the ARNT A, H and I strands as mutagenesis targets, but in this case the two AhR interfaces are dramatically different and involve different structural elements of AhR: the helical region in the *PASB*.*4F3L* model and the A and I strands in the *PASB*.*3F1P* model (Table 4).

All but one of the targeted mutations were designed to disrupt AhR:ARNT PAS domain dimerization. In the *PASA*.*4F3L* model, the mutations A119R in AhR, and L167E and I340R in ARNT would result in an insertion of a charged residue into the hydrophobic core of the PPI interface; conversely, the R236A mutant of AhR and E163A of ARNT may impair the formation of putative intermolecular salt bridges (Fig 6A); R342A of ARNT was designed to perturb the correct placement of the A’ helix. A unique mutation was planned to reinforce the interaction between the AhR H strand and the ARNT A’ helix: the Q234R mutant of AhR would extend the electrostatic network involving R236 in AhR and E170 in ARNT. In the *PASA*.*4M4X* model: F115S of AhR and Y311Q of ARNT is proposed to disrupt the stacking interaction between the A’ helix of the former protomer and the H strand of the latter; K238A of AhR and D161A of ARNT may impair the formation of a salt bridge between these two residues; F260S of AhR and L169R of ARNT would cause the insertion of a polar or charged residue into the hydrophobic environment (Fig 6B).

In the *PASB*.*4F3L* model: mutations within the bundle of aromatic residues that characterize the PPI interface (F289L and Y316R in AhR, F444E, F446S and Y450Q in ARNT) may disrupt the stacking interactions and/or insert polar or charged side-chains into this hydrophobic environment; I324R in AhR would place a charged residue directly in the middle of the same region (Fig 6D); in contrast, the ARNT mutants R366A, N448A and E455A may perturb the network of electrostatic interactions in the solvent exposed portion of the dimerization interface (Fig 6C). In the *PASB*.*3F1P* model: mutations K284D in AhR and E362K in ARNT may eliminate the putative salt bridge between these residues; both I374W of AhR and I458W of ARNT would augment the steric hindrance of the side-chain to perturb the region of hydrophobic interactions; F444R of ARNT would insert a charge residue in the same region (Fig 6E).

### Functional analysis of AhR and ARNT mutations

The proposed mutations (Table 4) were introduced into AhR and ARNT sequences using site-directed mutagenesis and the resulting mutant AhR and ARNT constructs were sequence verified. The introduced mutations did not affect protein expression of AhR or ARNT constructs in the reticulocyte *in vitro* transcription/translation (TNT) system (S8 Fig). The TNT experimental system has been extensively validated and utilized for more than 20 years to analyze the mechanisms of AhR activation and a major application has been for the analysis of ligand-induced AhR:ARNT dimerization and subsequent DRE-dependent DNA binding [4,7,19,38–40]. The ligand-dependent binding of *in vitro* synthesized AhR:ARNT complexes to DRE-containing DNA can be easily visualized by gel retardation analysis (S9 Fig). This sample gel demonstrates lack of DRE-bound complex with unprogrammed lysate, as well as the supershifting of the AhR:ARNT:[^32^P]DRE complex in the presence of anti-AhR and anti-ARNT antibodies, confirming the presence of these specific proteins in the induced protein-DNA complex.

The TNT expression system is derived from mammalian cells (rabbit reticulocytes) and has been shown to produce fully functional AhR capable of ligand-binding, hsp90 binding, ARNT dimerization and ligand-dependent DNA binding (as a dimer with ARNT) and allows for easy manipulation of primary protein sequence [4,7,19,38–41]. However, the TNT system does have some limitations, including that only a fraction of the expressed AhR protein appears to be functional (in contrast, ARNT appears to be fully functional). This may contribute to the elevated background activity observed in methods which rely on detection of labeled AhR protein, such as that of co-immunoprecipitation (CoIP). Indeed, CoIP experiments with *in vitro* synthesized radiolabeled AhR have revealed high background signal and a small signal/background ratio [4], making CoIP approaches problematic for clear and accurate qualitative and quantitative assessment of the functional activity of mutant and *wild-type* AhR/ARNT dimers. In contrast, both ligand binding (hydroxyapatite binding) and DNA binding (gel retardation analysis) methods rely on detection of [^3^H]TCDD- and [^32^P]DRE-binding to functional AhR, respectively. Accordingly, these latter experimental approaches have been shown to be valid and appropriate for quantitative assessment of AhR functional activities. Given the well-established overall correlation between AhR:ARNT dimerization and DNA binding by *wild-type* and mutant AhRs (except those mutations specifically targeting the DNA binding domain [41]), we analyzed the ligand-dependent AhR/ARNT activation using gel retardation analysis as an indirect measure of normal functional ligand-dependent AhR:ARNT dimerization. For those AhR mutations that result in significant reductions in ligand-dependent DNA binding, we also conducted [^3^H]TCDD ligand binding analysis in order to determine whether the reduction in DNA binding was actually due to a significant reduction in ligand binding ability or whether it was due to an alteration in DNA binding. Given that it has been well established that dimerization of ARNT with agonist-bound AhR is the critical event in AhR transformation (i.e. conversion of the AhR into its DNA binding form [4–7]), those AhR mutations that result in decreased ligand-dependent AhR:ARNT DNA binding but show little decrease in [^3^H]TCDD ligand binding must be adversely affecting transformation of the AhR into its normal functional DNA binding form. The most likely explanation for this alteration in AhR transformation is that the targeted mutation leads to an alteration in the normal protein-protein interactions important in normal functional dimerization between AhR with ARNT, leading to decreased AhR:ARNT DNA binding

**Fig 7:**
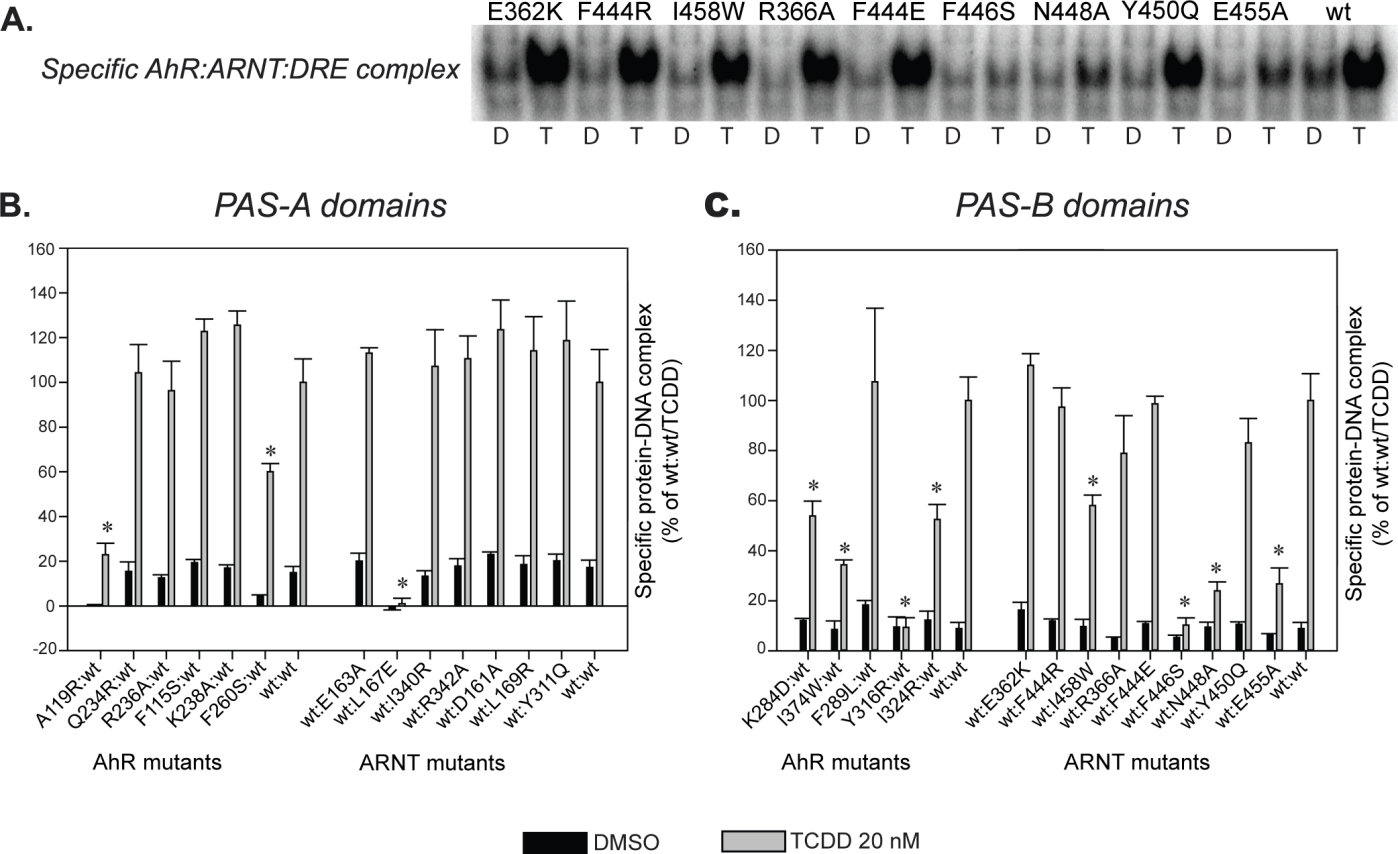
Selected point mutations of AhR and ARNT decrease PAS-A and/or PAS-B DNA-binding ability of ligand-activated AhR/ARNT dimer. The indicated AhR and ARNT mutant constructs were synthesized *in vitro*, transformed in the presence of *wild-type* dimerization partner and 20 nM TCDD (or solvent control DMSO), and analyzed by gel retardation analysis for DNA binding. (A) Representative gel is shown as an example of the effect of selected PASB ARNT mutations on ligand-dependent AhR:ARNT DNA binding. D, DMSO; T, TCDD. (B) and (C) Protein-DNA complexes in gels as in A were quantitated and the values corresponding to the intensity of the specific complex are expressed as mean +/- standard deviation of three replicate reactions. Those values statistically different from the *wild-type*/TCDD control at *p* < 0.05 (Student’s t-test) are indicated with an asterisk (*). All experiments were performed twice and the results were consistent between the runs.

DNA binding analysis of *in vitro* synthesized AhR and ARNT, incubated in the presence of 20 nM TCDD for 2 h prior to DNA binding (sample gel shown in Fig 7A), revealed several AhR and ARNT mutants with significantly decreased DNA binding activity compared to the response obtained with *wild-type* (wt) AhR and ARNT (Fig 7B and 7C). Mutant sets characteristics of either the PAS-A or PAS-B heterodimer models derived using the CLOCK:BMAL1 (4F3L) template (see Table 4) demonstrated both the higher number of affected mutants (7 out of 16 proposed 4F3L mutants affected or 50% rate; while with non-4F3L mutants 4 out of 11 affected or 36%), and more significant decreases in the level of AhR:ARNT DNA binding. In particular, the L167E PAS-A domain mutant of ARNT, the Y316R PAS-B domain mutant of AhR and F446S PAS-B domain mutant of ARNT, resulted in dramatically decreased levels of ligand (TCDD)-stimulated DNA binding, with respect to wtAhR and wtARNT (Fig 7B and 7C).

Given similar levels of *in vitro* expression of all AhR and ARNT mutant proteins (S8 Fig), the observed decreases in functional activity of a specific mutant ARNT protein would likely be due to an effect of the mutation on normal ligand-dependent AhR:ARNT dimerization as ARNT has no adverse effect on AhR ligand binding. All ARNT mutants with significantly decreased DNA binding levels were derived from the *PASA*.*4F3L* or the *PASB*.*4F3L* models (L167E in PAS-A, and F446S, N448A, and E455A in the PAS-B: all with an 80% or greater decrease in activity relative to wtARNT), and only one moderately decreased mutant ARNT (I458W, ~50% decrease in activity) was derived from the 3F1P model.

**Fig 8:**
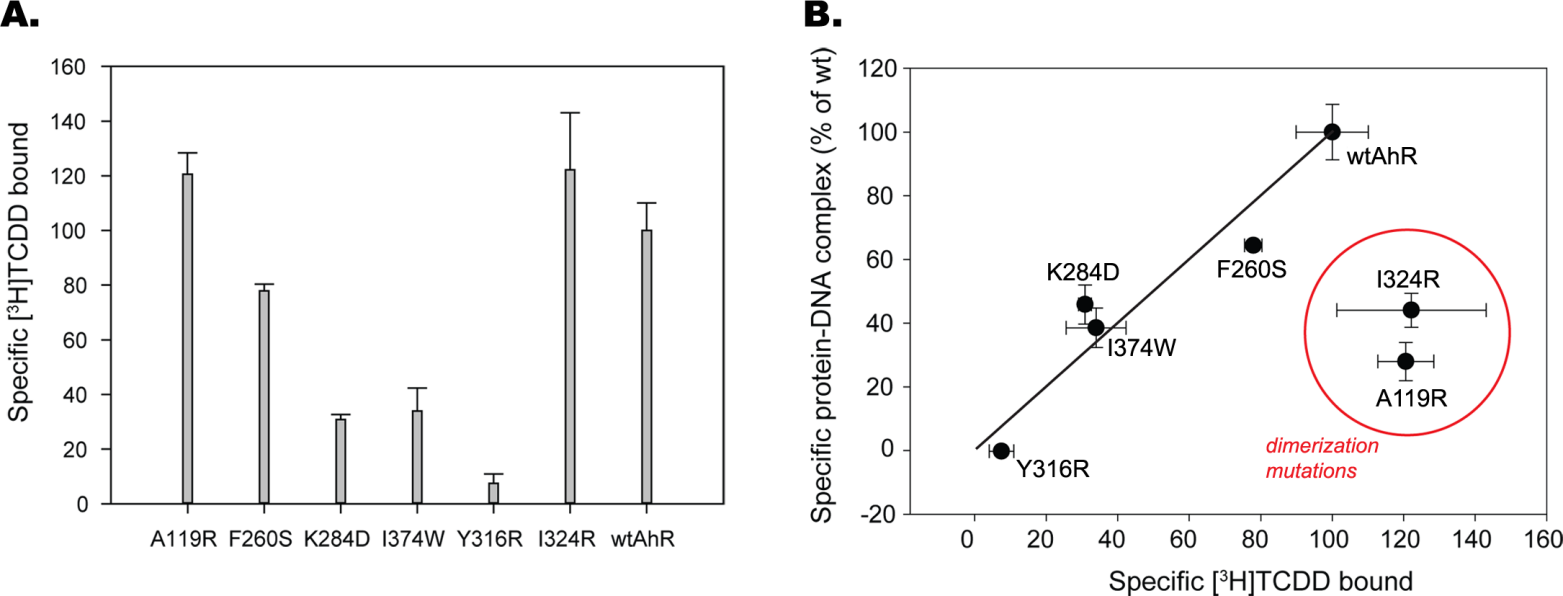
Selected AhR mutations demonstrate impaired ligand binding ability. (A) Indicated AhR mutants were synthesized *in vitro* and analyzed for [^3^H]TCDD ligand binding. Specific binding of [^3^H]TCDD to each mutant AhR was calculated as a difference of [^3^H]TCDD bound to *in vitro* reticulocyte lysate expressed AhR mutant and to that of unprogrammed lysate (background value) reactions. Values are expressed as mean +/- standard deviation of three replicate reactions. Mean values for all mutants were significantly higher than the background value at *p* < 0.05 as determined by the Student’s t-test. The results are representative of three independent experiments. (B) Values for specific protein-DNA complex formation for indicated mutants (y axis) were plotted against the respective values for specific [^3^H]TCDD binding. All values are expressed as mean +/- standard deviation of three replicate reactions and were normalized to values obtained with wt AhR (set at 100%). The line represents values that have a direct 1:1 relationship between ligand binding and DNA binding. Results with mutant AhRs that fall in the lower-right half of the plot and circled in red (A119R, I324R) indicate a likely effect of the mutation on normal ligand-dependent AhR/ARNT dimerization and thus DNA binding, as described in the main text.

In contrast, mutations of AhR may also impair its ligand-binding properties, especially in residues contained within the TCDD-binding fingerprint in the AhR PAS-B domain [19]. Accordingly, all AhR mutants that exhibited reduced ligand-dependent DNA binding activity (A119R and F260S in PAS-A and K284D, I374W, Y316R and I324R in PAS-B, Fig 7B and 7C) were further examined by [^3^H]TCDD ligand-binding analysis in order to determine whether the introduced mutations adversely affected AhR ligand binding and thus the ability of AhR to transform into a form that can functionally dimerize with ARNT and subsequently bind to DNA. Ligand binding analysis demonstrated that several AhR mutants including F260S, K284D, I374W and Y316R exhibited reduced [^3^H]TCDD binding ability relative to that of the wtAhR (Fig 8A). Three of these mutants were derived from the *PASA*.*4M4X* and *PASB*.*3F1P* models, and therefore the observed decrease in DNA binding ability of these mutant AhRs is likely due to their impaired ligand-binding ability.

This point can be better illustrated by comparing specific DNA binding versus specific ligand binding for mutant AhRs (Fig 8B). In this graph, data points for AhR mutants F260S, K284D, I374W and Y316 are located near the line indicating that these AhRs exhibit the same relative ratio of ligand binding to DNA binding, but the ligand-binding and DNA-binding activity of these mutant AhRs were proportionally decreased relative to that of the wtAhR. These results suggest that these mutations simply affect the AhR in a manner that reduced its ligand binding activity and thus its relative ability to be converted into a form that can dimerize with ARNT and bind to DNA, without differentially affecting AhR:ARNT dimerization interactions. Previous mutagenesis studies demonstrating that mutation of Y316 decreased both AhR ligand and DNA binding [19] are consistent with this conclusion. In contrast, while the 4F3L-derived mutants (A119R and I324R) demonstrated no reduction in [^3^H]TCDD specific binding, they exhibited significantly reduced ligand-dependent DNA binding, consistent with impaired dimerization as a likely underlying mechanism.

When the effect of decreased functional activity in AhR mutants F260S, K284D and I374W is factored into the overall analysis, only 1 out of 11 (or 9%) non-4F3L model-derived mutants demonstrated a moderate decrease in DNA binding (I458W), while 7 out of 16 of the 4F3L-derived mutants (45%) demonstrated decreased DNA binding. Taken together, these data are more consistent with the 4F3L-derived heterodimerization models as being the more reliable ones for the AhR:ARNT dimerization mode of both PAS-A and PAS-B domains.

## Discussion

The computational approaches developed in this work allowed generation of alternative AhR:ARNT PAS-A and PAS-B dimer models and provided framework to analyze the molecular determinants and their contribution to dimer stability in each model. Despite the high sequence identities and similarities with all the templates adopted, the analysis of the physico-chemical complementarity of the residues typical of the AhR:ARNT PAS dimerization interfaces allowed to predict that the AhR:ARNT PAS-B model based on the CLOCK:BMAL1 template [13] (*PASB*.*4F3L*) is more reliable than the one based on the HIF2α:ARNT complex [11] (*PASB*.*3F1P*). In contrast, the two alternative PAS-A dimer models showed similar dimerization interfaces, and no conclusive preference of one model over another emerged based on computational studies alone. However based on these computational analyses, the subsequent site-directed mutagenesis plan was developed for both PAS-A and PAS-B dimer models and functional analysis was performed for experimental validation of the optimal model. The results of functional analysis of the targeted AhR and ARNT mutations derived from modeling of AhR:ARNT interactions strongly support the CLOCK:BMAL1-derived dimer models for both PAS-A and PAS-B domains.

**Fig 9:**
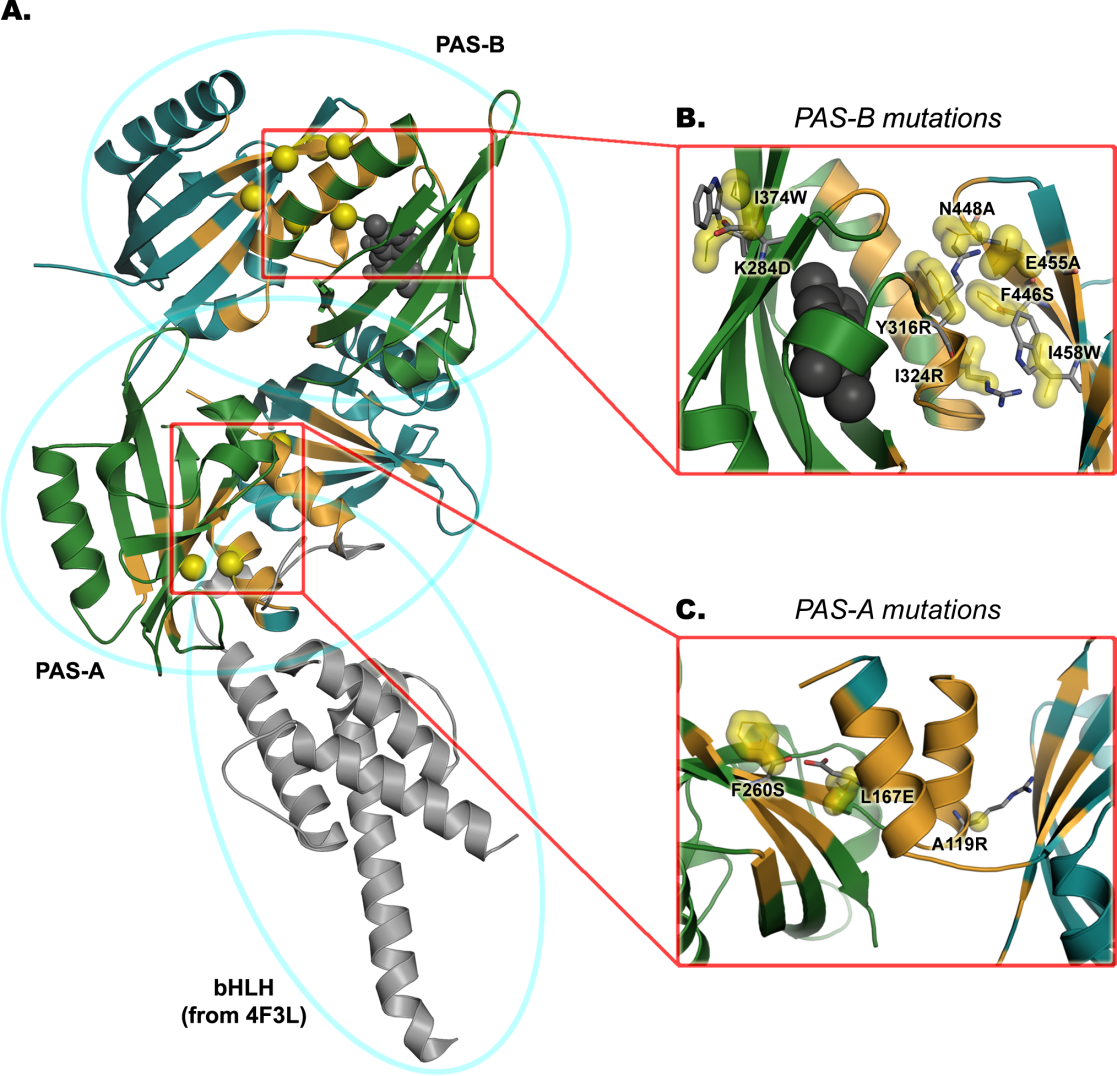
Overall representation of the PASA.4F3L and PASB.4F3L and related mutations that decrease DNA binding activity. AhR is colored in green, ARNT in cyan, the PAS dimerization interfaces in orange, the positions of the mutated residues in yellow, the TCDD ligand is represented in dark grey spheres. (A) The PAS dimer models are superimposed to the full-length 3D scaffold of the CLOCK:BMAL1 template; the orientation of the bHLH motif (highlighted in grey) is taken from the experimental atomic coordinates of the 4F3L crystallographic structure. (B) Mapping of the mutations along the *PASB*.*4F3L* dimer model. The coordinates of the *wild-type* residues are highlighted in yellow, putative rotamers of the mutated residues are shown in grey sticks. (C) Mapping of the mutations along the *PASA*.*4F3L* dimer model.

The overall representation of the modeled dimer structures, superimposed on the full-length 3D scaffold of the CLOCK:BMAL1, is given in Fig 9A.

In the PAS-A region, the general characteristics of the PPI interfaces of both the AhR:AhR and the CLOCK:BMAL1 templates are maintained, but the orientation of the A' α-helices with respect to the PAS cores more strictly resembles that of CLOCK:BMAL1 (see also S2A Fig). In the PAS-B dimer, the helical bundle of AhR interacts with the β-sheet of ARNT (as observed between the CLOCK helices and the BMAL1 β-sheet in the template structure, see also S2B Fig).

In the PAS-A region, two mutations were shown to dramatically decrease the levels of DNA binding: AhR:A119R and ARNT:L167E (Fig 7B). Both of these mutations introduce a charged residue into the hydrophobic environment delimited by the A’ α-helix and the β-sheet of the opposite protein partner (Fig 9C). Both mutations were designed to selectively disrupt the *PASA*.*4F3L* dimer model, since in the *PASA*.*4M4X* model these residues are not important for dimer stabilization and are located at the boundary of the dimerization interface, where they are oriented outward into the solvent exposed region.

The residue AhR:A119 was previously mutated as AhR:A119D for the characterization of the murine AhR homodimer (4M4X) [12] and appeared critically important, since its mutation affected AhR PAS-A dimerization. The authors inferred, on the basis of their model of the AhR:ARNT PAS-A dimer, that this mutant would also influence the AhR:ARNT dimerization.

In our work, AhR:A119 is predicted to be part of the Δ*G signature* of the *PASA*.*4F3L* model (Fig 3A) indicating that its stabilizing contribution would be higher in this model than in the *PASA*.*4M4X* model. Moreover, in the *PASA*.*4M4X* model the residue shows an individual contribution to ΔSASA [27] that is only 35% of that in the *PASA*.*4F3L* model, indicating that AhR:A119 is more buried into/interactive at the dimerization interface of the *PASA*.*4F3L* model, and therefore disruption of dimerization with mutations at this position, observed in this study and previously [12], preferentially lends support to the *PASA*.*4F3L* model.

A third mutant in the PAS-A domain, AhR:F260S, designed to disrupt dimer formation in the *PASA*.*4M4X* model with a polar residue in the hydrophobic environment, decreased the specific AhR:ARNT DNA binding activity to about 70% of that of wtAhR:ARNT (Fig 7B). A similar mutation (AhR:F260D) was previously reported for the AhR homodimer–even though it was not fully characterized due to technical difficulties–and it showed weak effect on the AhR activity, as assessed by the XRE luciferase reporter assay [12]. Our ligand binding analysis demonstrated that AhR:F260S also reduced [^3^H]TCDD specific binding relative to that of the wtAhR (Fig 8) and this could contribute to the observed decrease in DNA binding. It is unclear why this mutation, located in the AhR PAS-A and outside the ligand-binding domain PAS-B, results in decreased functional activity of the AhR. It is possible that AhR:F260S may affect AhR:hsp90 interactions and/or interfere with inter-PAS-domain geometry in the AhR, resulting in overall reduced functional activity. However, since the effects of this mutation did not appear to be directly related to AhR:ARNT dimerization, given the somewhat proportional decreases in both ligand binding (to ~80% of wtAhR) and DNA binding (to ~70% of wtAhR), the functional data obtained with this *PASA*.*4M4X*-derived mutant should not be considered in ranking PASA dimerization models.

Among the panel of mutations proposed for the PAS-A domains, the residues ARNT:D161 and ARNT:E163 were previously considered by other authors [35], who planned a panel of mutations specific for the interaction between the PAS-A domains of AhR and ARNT, without prior knowledge of experimental 3D structures of PAS-A dimers in bHLH/PAS proteins. Indeed, most of the mutations in this previous study map significantly outside any credible PPI dimerization interface (as analyzed in our study on published 3D structures of PAS dimers [12,13]) and the observed effects were probably due to allosteric/concerted effects that would not directly involve the dimerization interfaces. In particular, this study reported the ARNT:D161G and ARNT:E163K mutations, which were dimerization-neutral and strongly dimerization-disruptive, respectively. The ARNT:D161A and ARNT:E163A mutations proposed in our work were designed to disrupt putative salt bridges in our PAS-A dimer models, however, no adverse effect on dimerization was observed (Fig 7B). Nevertheless, the dimer-disrupting effect of the mutation ARNT:E163K [35] further confirms the reliability of the *PASA*.*4F3L* model. The ARNT:E163 residue was in fact predicted as distinct PPI *hot spot* for this model (Fig 6A) and it is conceivable that while the mutation to alanine does not critically affect the dimer stability, the ARNT:E163K could introduce a sufficiently repulsive electrostatic effect into the putative salt bridge with AhR:K238 to interfere with the dimerization process.

Among mutations in the PAS-B domains, those indicative of the dimerization interface of the *PASB*.*4F3L* dimer model clearly showed disrupting effects in the functional assay. Specifically, the F446S, N448A and E455A mutants of ARNT strongly decreased the DNA binding activity of the ligand-activated AhR:ARNT dimers (Fig 7C). A possible explanation is that ARNT:F446S introduces a polar residue into a highly hydrophobic region and disrupts putative stacking interaction between aromatic residues, whereas N448A and E455A perturb the network of electrostatic interactions at the solvent exposed portion of the dimerization interface (Fig 9B). On the other hand, an ARNT mutant (I458W) designed to introduce steric hindrance in the *PASB*.*3F1P* interface also resulted in a moderate decrease in DNA binding, the only mutant to do so in the *PASB*.*3F1P* derived set (Fig 7C). Since this residue is located close to the *PASB*.*4F3L* PPI hydrophobic core (Fig 9B) it is conceivable that a tryptophan side-chain in this region may perturb the hydrophobic core and interfere with dimerization. A similar effect was observed with a mutation in the topological equivalent position of ARNT:I458 in the CLOCK:BMAL complex (V435R in [13]), but in that case a charged residue (arginine) was inserted.

Mutation of several PAS-B AhR residues were shown to affect AhR:ARNT DNA binding (Fig 7C). Y316R and I324R were designed to introduce a charged residue into the hydrophobic environment in the *PASB*.*4F3L* model, while I374W and K284D were designed to perturb the *PASB*.*3F1P* dimer by introducing steric hindrance in the interface and by disrupting the putative salt bridge with ARNT:E362, respectively. All AhR PAS-B mutations that affected AhR:ARNT DNA binding were subjected to [^3^H]TCDD ligand binding analysis to determine if they affected the AhR ligand-binding properties. These results confirmed that while the I324R mutation had no effect on AhR ligand binding, it significantly reduced AhR:ARNT DNA binding, consistent with an effect on AhR:ARNT dimerization (Fig 8B). The weaker disrupting effect, compared to the other disrupting mutations (about 60% of the wtAhR:ARNT DNA binding activity), could be due to the conformational flexibility at the position I324, that allows the mutated arginine to point outside the interface and be partially stabilized by the aqueous external environment. Mutations to hydrophobic residues at this position did not significantly affect ligand or DNA binding activity (I324A [75% and 100%, respectively] and I324M [90% and 100% of wt, respectively]).

In contrast, the ligand binding analysis showed that the AhR:Y316R mutation reduced [^3^H]TCDD binding, a result consistent with previous studies that included Y316 in the TCDD-binding fingerprint of mammalian AhRs [19]. Y316 is located in the flexible region of the C-E helices and the lining of the modeled ligand binding cavity, and when mutated to alanine it resulted in the loss of AhR ligand and DRE binding, while mutation to phenylalanine moderately decreased levels of ligand binding (45%) and DRE binding (84%) [19]. Together these results suggest that this residue may not only impact ligand binding, but also AhR transformation and/or AhR:ARNT dimerization/DNA binding. The key position on the border between the ligand-binding cavity and the dimerization interface renders the Y316 residue critically important and sensitive to any chemical/structural perturbation. Further studies are needed to elucidate its role in the AhR activation mechanism induced by ligands.

The TCDD binding assay results clearly show how the mutations of AhR derived from the *PASB*.*3F1P* dimer model (i.e., AhR:I374W and AhR:K284D) impair ligand binding instead of dimerization and DNA binding (Fig 8B). Their role in TCDD binding may be explained based on the proximity to AhR:A375 and AhR:H285, that point their side-chains into the ligand binding cavity and are well known determinants of TCDD binding within the AhR ligand binding pocket [19]. The fact that these mutations, mapping on the opposite side of the dimerization interface of the *PASB*.*4F3L* dimer model (Fig 9B), do not affect dimerization confirms the applicability of the CLOCK:BMAL1 dimerization mode for AhR:ARNT dimerization model.

The topologically equivalent position of AhR:I374 was previously investigated in the homologous dimer HIF2α:ARNT (mutation HIF2α:M338E in [37]). In that study, the mutation maps on the dimerization interface involving the β-sheets of both the HIF2α an ARNT partners and the observed negative effect was due to dimerization disruption, since activation of HIF2α is not dependent on ligand binding. Similarly, the mutations AhR:K284D and ARNT:E362K map to positions previously investigated on the homologous complex HIF2α:ARNT [11], in which these amino acids were located in the β-sheets at the dimer interface. In that case, the authors prepared a double mutant HIF2α:R247E/ARNT:E362R in order to reinforce a pre-existing salt bridge and to stabilize the heterodimer complex. While the double mutants indeed resulted in a more stable complex, the single mutants showed 3- to 5-fold lower affinities than the *wild-type* complex. These results together with our data refuting the HIF2α:ARNT heterodimer-based *PASB*.*3F1P* model further suggest different dimerization modes of AhR and HIF2α and support the reliability of the proposed CLOCK:BMAL1-based AhR:ARNT dimer model.

### Concluding remarks

Aim of this paper was to unveil the structural mode of dimerization of AhR:ARNT and identify the essential interacting interfaces in both the PAS-A and PAS-B domains. Our studies suggest for the first time that the AhR and ARNT PAS domains have dimerization modes similar to those observed in CLOCK:BMAL1. In addition to a number of computational analyses on the alternative dimer models developed, the reliability of the proposed structures as well as their functional relevance are strongly supported by our site-directed mutagenesis and functional analysis experiments. The very recently published structures of the HIF2α:ARNT complex including the entire bHLH-PASA-PASB domains [42], further confirm our findings. In fact, both the PAS-A:PAS-A and the PAS-B:PAS-B interdomain interfaces in these new structures are very similar to those observed in the CLOCK:BMAL1 heterodimer [13]. It is conceivable that the dimerization mode previously observed for the isolated PAS-B domains of HIF2α and ARNT [8–11] is less functionally relevant than the modes observed upon crystallization of the entire bHLH-PASA-PASB region in the same complex as well as in CLOCK:BMAL1.

Our results suggest that, not only structures, but also the dimerization modes of the PAS domains are well conserved in bHLH-PAS proteins. However, the functional roles and the biochemical mechanisms of action of proteins belonging to this family are distinctly different. In particular, since AhR is the only bHLH-PAS protein characterized by a ligand-activated mechanism, our models could be the starting point for unraveling the key aspects of a mechanism that could not be investigated at a molecular level until now.

In particular, the results reported here combined with the availability of the experimentally resolved scaffold of the entire N-terminal portion of two bHLH-PAS dimers [13,42], offer an unprecedented opportunity to build a full-length model of the N-terminal region of the AhR:ARNT complex. Given that different quaternary architectures are suggested by the two templates for this region [42], only an accurate modeling of the structure and dynamics of the regions connecting the AhR and ARNT domains combined with experimental validations would allow to propose the more appropriate structure for AhR:ARNT and to characterize the critical PPI hallmarks in the intra- and inter-domain interfaces. These studies would provide important new insight into the role of inter-domain interactions in the signal transmission, from ligand activation to the conversion of the AhR into its high-affinity DNA binding form. Moreover, structural insights here derived for the PAS domain interactions of AhR and ARNT could lead to an increased understanding of the interactions of these domains with other partners, such as the chaperone hsp90, the AhR repressor, as well as that of co-activator proteins.

Detailed characterization of protein-protein interactions, both inter-domain (within the AhR protein) and with AhR interacting proteins, would open new avenues to analyze physiological roles of the AhR including both the classical and non-classical mechanisms of its activation.

## Methods

### Homology modeling

PAS-A and PAS-B domain structural models were built for the murine isoforms of the AhR and ARNT proteins (mAhR: UniProt Q3U5D9, GI 123784256; mARNT: UniProt P53762, GI 341940591). To obtain reliable alignments between the target sequences and the structural templates, both the sequences and the secondary structure (SS) profiles were taken into account. For the target sequences the SS profile was predicted by using the PSIPRED webserver [43,44] while for the templates it was attributed by means of the DSSPcont algorithm [45]. The models were built using the MODELLER 9v8 software [18]. From each alignment 100 putative models were produced and ranked according to the DOPE distance-dependent statistical potential [46]. The overall quality of the best-ranked models was assessed with PROCHECK [22], which provides information about the stereo-chemical quality, and ProSA [23,24] validation method, which evaluates model accuracy and statistical significance with a knowledge-based potential.

Several residues were not solved in the PAS-A experimental structures: in the AhR (4M4X), residues 174–209 and 245–252 (in the FG and HI loops respectively); in ARNT (4F3L), residues: 228–259, 272–301 and 315–334 (in the FG, GH and HI loops, respectively). Based on the highly conserved structural folds of the PAS-A and PAS-B domains (RMSD values for the Cα atoms in the core region: 1.31 Å between hARNT.PAS-B (3F1P) and mBMAL1.PAS-A (4F3L); 1.44 Å between the mAhR PAS-B homology model [17] and PAS-A (4M4X)), these missing regions were filled by grafting the atomic coordinates of the topologically equivalent PAS-B loops onto the PAS-A structures. The overall quality of the PAS-A models and the influence of the inserted loops were evaluated through the inspection of the related DOPE profiles.

### Energy minimization

To remove residual steric clashes and relax the overall structure of the dimer models, energy minimization protocol was performed using the AMBER software [47]. The protein systems were described by the AMBER force field 99SB [48], whereas the topology of the TCDD ligand, that was included in the mAhR PAS-B model, was treated with the general AMBER force field (GAFF) [49], with atomic partial charges evaluated with the semiempirical method AM1-BCC [50]. The models were placed in a periodic truncated octahedron box with a minimum distance of 10 Å from the protein surface. The systems were solvated using the TIP3P water model [51] and the net charges were neutralized by adding counterions. The minimization was performed by using 500 steps of steepest descent, followed by 1500 steps of conjugate gradient. Van der Waals and short-range electrostatic interactions were estimated within a 8 Å cutoff, whereas long-range electrostatic interactions were calculated using the Particle Mesh Ewald method [52]. The ligand and the backbone atoms of the protein were restrained with a restraint of 120 kcal mol^-1^ Å^-2^.

### Molecular Mechanics Generalized Born Surface Area (MM-GBSA)

The binding free energy (Δ*Gbinding*) of each modeled dimer was evaluated through the MM-GBSA method. In this context the “ligand” is the ARNT and the “receptor” is the AhR protomer.

The MMPBSA.py module of the AMBER software was adopted, with the single protocol strategy [53] (i.e. calculations were performed starting from an optimized structure of each model instead of an ensemble of snapshots sampled from Molecular Dynamics simulations). The input structures were treated in implicit solvent [54]. The polar solvation term was approximated with the Generalized Born (GB) model [55] by using the OBC re-scaling of the effective Born radii [56]. A physiological salt concentration (0.154 M) was chosen to take into account the electrostatic screening effect of salt [57]. The non-polar solvation term was calculated as the product of the surface tension parameter, *surften* (set to 0.0072 kcal mol^-1^ Å^-2^), and the solvent accessible surface area (*SA*) evaluated using the Linear Combination of Pairwise Overlap (LCPO) algorithm [58].

The Δ*Gbinding* calculated by the MM-GBSA method was used for the Energy Decomposition Analysis [29,30] of each dimer, as detailed in the S1 Appendix. To this end, for each system (i.e. the receptor, the ligand and the complex) the free energy was decomposed into the residue pairwise contributions and for each pair it was further decomposed into the different terms (covalent, electrostatic, van der Waals and solvation). Every energetic contribution was subsequently processed as:

$$\Delta G_{ij}=G_{ij}^{complex}-\left(G_{ij}^{receptor}+G_{ij}^{ligand}\right)$$

where *G* is the specific term taken into account, *i* and j are the specific residues involved in the pairwise interaction. Given that in the MM-GBSA approach here employed both the receptor and the ligand conformations are extracted from the optimized geometry of the complex, all the intramolecular residue pairs give Δ*G*_*ij*_ = 0. The sum of the non-bonded and the solvation terms for each intermolecular residue pair defines its individual contribution to the binding free energy. All of such contributions are represented as a matrix, herein defined as *interaction energy matrix*.

The relative per-residue contributions to the overall binding energy of the dimer models were compared by means of the Rank Products algorithm [28,29], as detailed in S1 Appendix. A necessary assumption is that two dimer models subjected to comparison are assembled from the same protomer models that can be oriented differently, resulting in alternative dimerization interfaces. Considering two hypothetical dimers A and B, each residue *g* of the protomer is ranked on the basis of its relative contribution to the Δ*Gbinding*, thus generating two distinct scores *R*_*g*_^A^ and *R*_*g*_^B^. The difference between the scores indicates if residue *g* mostly contributes to dimer A (positive values) or B (negative values). Results are presented in logaritmic form, LOG(*RP*). The subset of LOG(*RP*) values with the same sign indentifies the minimal lists of residues that differentially contribute to Δ*Gbinding* of each dimer model, and it is herein termed Δ*G signature*.

### PPI interface characterization and hot spot collection

The dimerization interface of each dimer model was analyzed using PISA (Proteins, Interfaces, Structures and Assemblies) web server [25]. The resulting list of residues included in the interface was investigated in terms of possible intermolecular non-bonded interactions (namely, van der Waals and H-bonds) using the DIMPLOT module, a specific extension of LigPlot+ software for plotting protein-protein or domain-domain interactions [26]. The burial degree of the individual domains upon complexation was evaluated through the calculation of the variations of the Solvent Accessible Surface Area (ΔSASA) using NACCESS [27]. The Electrostatic Potential Surface (EPS) of each domain in the modeled dimers was calculated using the DelPhi v4 software [31], with a grid spacing of 0.5 Å.

*Hot spots* are defined as those residues belonging to a PPI interface whose sidechains are predicted to significantly stabilize the binding mode [59,60] and three different methods for predicting the *hot spots*, based on alternative methodological assumptions, were selected following previously reported comparative studies [61]. The predictions provided with the HotPoint method [32] are based both on evaluation of ΔSASA and on a scoring function termed *potential contact*, based on the number of interacting residues in a shell of 7 Å. With this method, a *hot spot* is found if the SASA is reduced at least by 20% and the potential contact score is at least 18. The predictions of the KFC2 method [33] are based on a machine learning method trained on 47 different features derived from solvent accessibility and biochemical properties of the residues (e.g., hydrophobic profiles, non-bonded interactions and π-stacking interactions). The predictions provided by the Robetta method [34] are obtained by performing *in silico* alanine scanning and calculating the ΔΔ*G* upon mutation with an internal energy function based on rotamers evaluation. *Hot spots* are defined here as those residues showing a ΔΔ*G* equal or larger than 1 kcal mol^-1^.

### *In vitro* functional analysis of AhR and ARNT mutants

Plasmids mβAhR/pcDNA3 and mβARNT/pcDNA3 have been previously described [7]. The accession number for the specific AhR cDNA is NM_001314027.1 and the accession number for the specific ARNT isoform a cDNA is NM_001037737.2. The point mutagenesis was performed using Quikchange Lightning Kit (Agilent) and the resulting plasmid constructs were verified by sequencing. To determine protein expression, constructs were synthesized in vitro using TNT Reticulocyte Lysate Kit (Promega) in the presence of [^35^S]methionine (Perkin-Elmer), separated on 4%-10% SDS-PAGE and visualized by autoradiography as previously described [62]. Gel retardation analysis of ligand-activated TNT-synthesized AhR and ARNT has been extensively documented in the literature [4,7,19,38–40] and is a well-established and validated method for analysis of AhR functionalities, including ligand binding, dimerization and DNA binding. For gel retardation analysis, AhR and ARNT proteins were synthesized in vitro using TNT Reticulocyte Lysate Kit (Promega), diluted (1:1:8, AhR:ARNT:buffer) in MEDGK (25 mM MOPS-NaOH, pH 7.5, 1 mM EDTA, 1 mM DTT, 10% [v/v] glycerol, 0.15 M KCl) and incubated in the presence of 20 nM TCDD or 1% (v/v) solvent control DMSO for 2 h prior to gel retardation analysis [62]. Briefly, AhR transformation reactions (10 ml) were incubated with 15 ml oligo buffer (41.7 mM MOPS-NaOH, pH 7.5, 16.7% [v/v] glycerol, 253 mM KCl, 16.7 mM DTT, 8.3 mM EDTA, 0.375 ng/ml poly [d(I•C)] [Roche]) for 15 min, followed by incubation with 100,000 cpm [^32^P]DRE for 15 min and gel separation on a 4% native polyacrylamide gel [40]. Gels were visualized by autoradiography using Fujifilm FLA-9000 and MultiGauge analysis. For ligand-binding analysis, in vitro synthesized AhR protein was diluted with MEDGK (1:9) and incubated in the presence of 10 nM [^3^H]TCDD for 1 h at room temperature followed by hydroxyapatite analysis as previously described [62]. Unprogrammed TNT lysate was used as non-specific control in binding reactions. [^3^H]TCDD was a kind gift from Dr. Stephen Safe (Texas A&M University).

## Supporting Information

S1 Fig The canonical fold of a PAS domain. In red are depicted the α-helices, in yellow the β-strands, in green those connecting loops whose length differs between the PAS-A and PAS-B domains (for such loops no structural information is available from the crystallographic templates of the PAS-A domains). The SS elements are labeled according to the nomenclature generally adopted for the PAS structures. In the figure the model of the mARNT PAS-A domain is shown.
(TIF)

S2 Fig Structural superposition of the templates adopted. (A) PAS-A dimers, the reciprocal spatial orientation of the A’ α-helices is highlighted. (B) PAS-B dimers.
(TIF)

S3 Fig Distance dependent statistical potential (DOPE) profiles. The protomer models of PAS-A domains (blue lines) are compared with the corresponding templates adopted (red lines). The green boxes highlight those regions that are not experimentally resolved in the structure of the template.
(TIF)

S4 Fig Sequence alignments between the murine AhR/ARNT PAS domains and the templates chosen. The lower case gray shaded residues highlight the regions that needed a refinement during modeling. The heading bars depict the secondary structure assignment according to PSIPRED prediction for the target sequence (mAhR/ARNT) or DSSPcont attribution over the structure of the templates. The light grey bars highlight the α-helices, the dark grey bars highlight the β-strands. Residues belonging to the PPI interfaces of both the models and the templates, as predicted by PISA, are highlighted by yellow boxes.
(TIF)

S5 Fig Distribution of the Z-score, calculated with ProSA. The distribution is represented along the sequence lengths taken from a dataset of experimentally resolved 3D protein structures. All of the models presented in this work (represented as black, yellow, green and red dots) fall into such distribution.
(TIF)

S6 Fig Electrostatic Potential Surface (EPS) of the PAS-A dimers, comparison between models and templates. In each panel, the individual domains constituting the dimers are rotated each other of 180 degree, to obtain a representation as an “open book”. The potential range is defined in kT/e units according to the DelPhi software. Only the region defining the dimerization interface is colored according to the potential scale. The main residue contacts in the models (Fig 6) as well as the topological equivalent positions in the templates are labeled. (A) *PASA*.*4F3L* dimer model and CLOCK:BMAL1 template. (B) *PASA*.*4M4X* dimer model and AhR:AhR template.
(TIF)

S7 Fig Electrostatic Potential Surface (EPS) of the PAS-B dimers, comparison between models and templates. In each panel, the individual domains constituting the dimers are rotated each other of 180 degree, to obtain a representation as an “open book”. The potential range is defined in kT/e units according to the DelPhi software. Only the region defining the dimerization interface is colored according to the potential scale. The main residue contacts in the models (Fig 6) as well as the topological equivalent positions in the templates are labeled. (A) *PASB*.*4F3L* dimer model and CLOCK:BMAL1 template. (B) *PASB*.*3F1P* dimer model and HIF2α:ARNT template.
(TIF)

S8 Fig Representative gels from mutant AhR proteins. Indicated mutant AhR proteins were expressed in vitro in the presence of [35S]-methionine and resolved on SDS-PAGE gel and visualized by FLA9000 PSL (phospho-stimulated luminescence) analysis.
(TIF)

S9 Fig Gel retardation analysis. Mouse AhR and ARNT were separately synthesized in vitro using TNT lysate, transformed in the presence of 20 nM TCDD or solvent control DMSO (1% v/v) and analyzed for DNA binding with the EMSA assay. Where indicated, 400 ng of anti-AhR antibody (M20), anti-ARNT antibody (N19) or corresponding IgG control (all antibodies and controls from Santa Cruz Biotechnology) were added to the DNA-binding reaction. In the unprogrammed lysate (UPL) reactions, no plasmid DNA was included during the in vitro synthesis step resulting in the absence of AhR or ARNT protein.
(TIF)

S1 Table Sequence identity and similarity between the dimer models and the templates adopted. (PDF)

S2 Table Overall quality indices of the modeled structures. (PDF)

S3 Table Rank Products profile for the PAS-A dimer models. (PDF)

S4 Table Hot spot list from PAS-A dimer templates, with related scores from the PPI prediction tools herein adopted (PDF)

S5 Table Rank Products profile for the PAS-A dimer models. (PDF)

S6 Table Hot spot list from PAS-B dimer templates, with related scores from the PPI prediction tools herein adopted (PDF)

S1 Appendix Energy Decomposition analysis and Rank Products algorithm. (PDF)
