# Supplementary figures and images for: Deciphering Dimerization Modes of PAS Domains: Computational and Experimental Analyses of the AhR:ARNT Complex Reveal New Insights Into the Mechanisms of AhR Transformation
Source: PLoS Comput Biol. 2016 Jun 13;12(6):e1004981. doi: 10.1371/journal.pcbi.1004981 (PMC4905635; doi:10.1371/journal.pcbi.1004981)

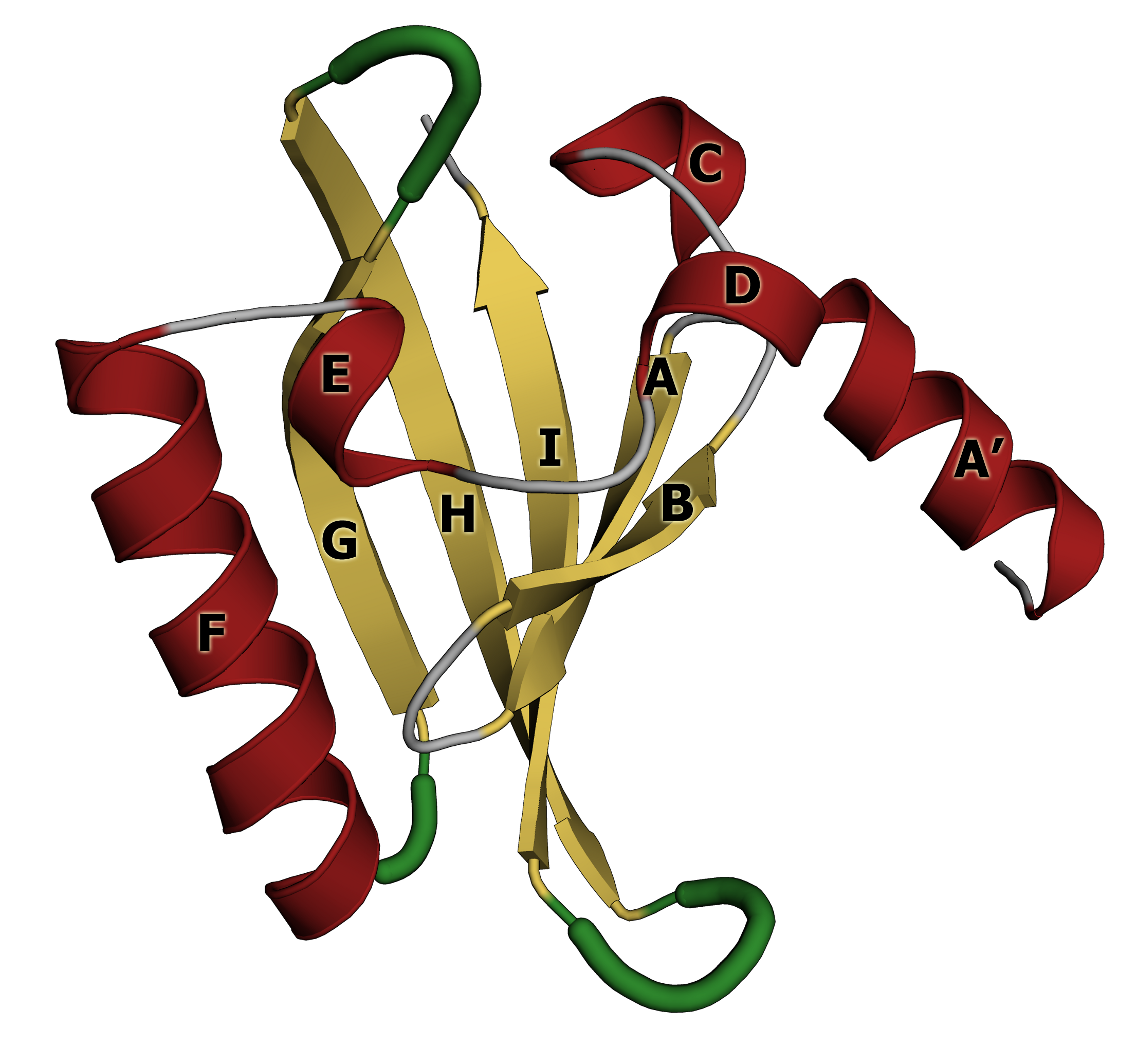

Supplement: S1 Fig — In red are depicted the α-helices, in yellow the β-strands, in green those connecting loops whose length differs between the PAS-A and PAS-B domains (for such loops no structural information is available from the crystallographic templates of the PAS-A domains). The SS elements are labeled according to the nomenclature generally adopted for the PAS structures. In the figure the model of the mARNT PAS-A domain is shown. (TIF) [file pcbi.1004981.s001.tif]

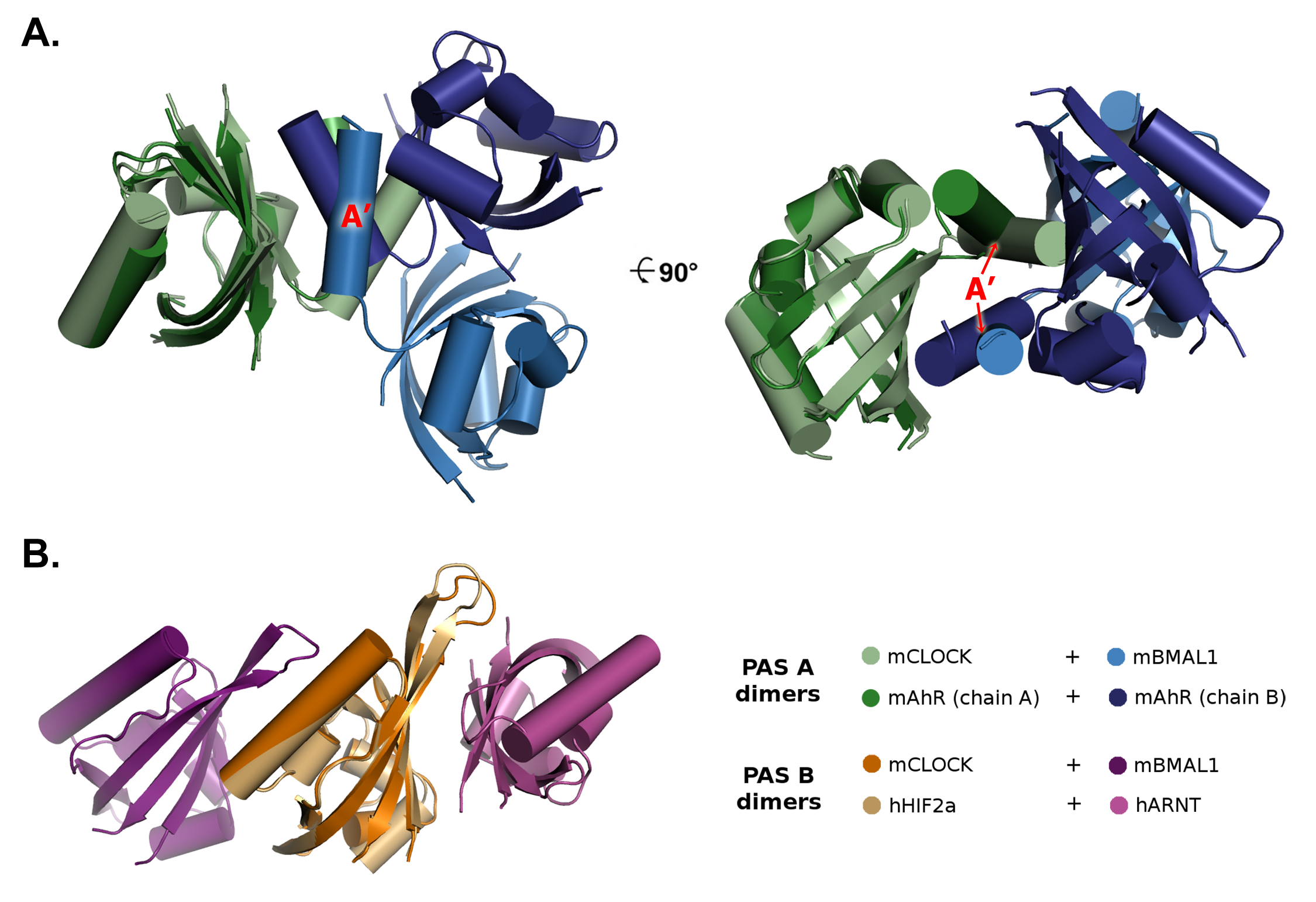

Supplement: S2 Fig — (A) PAS-A dimers, the reciprocal spatial orientation of the A’ α-helices is highlighted. (B) PAS-B dimers. (TIF) [file pcbi.1004981.s002.tif]

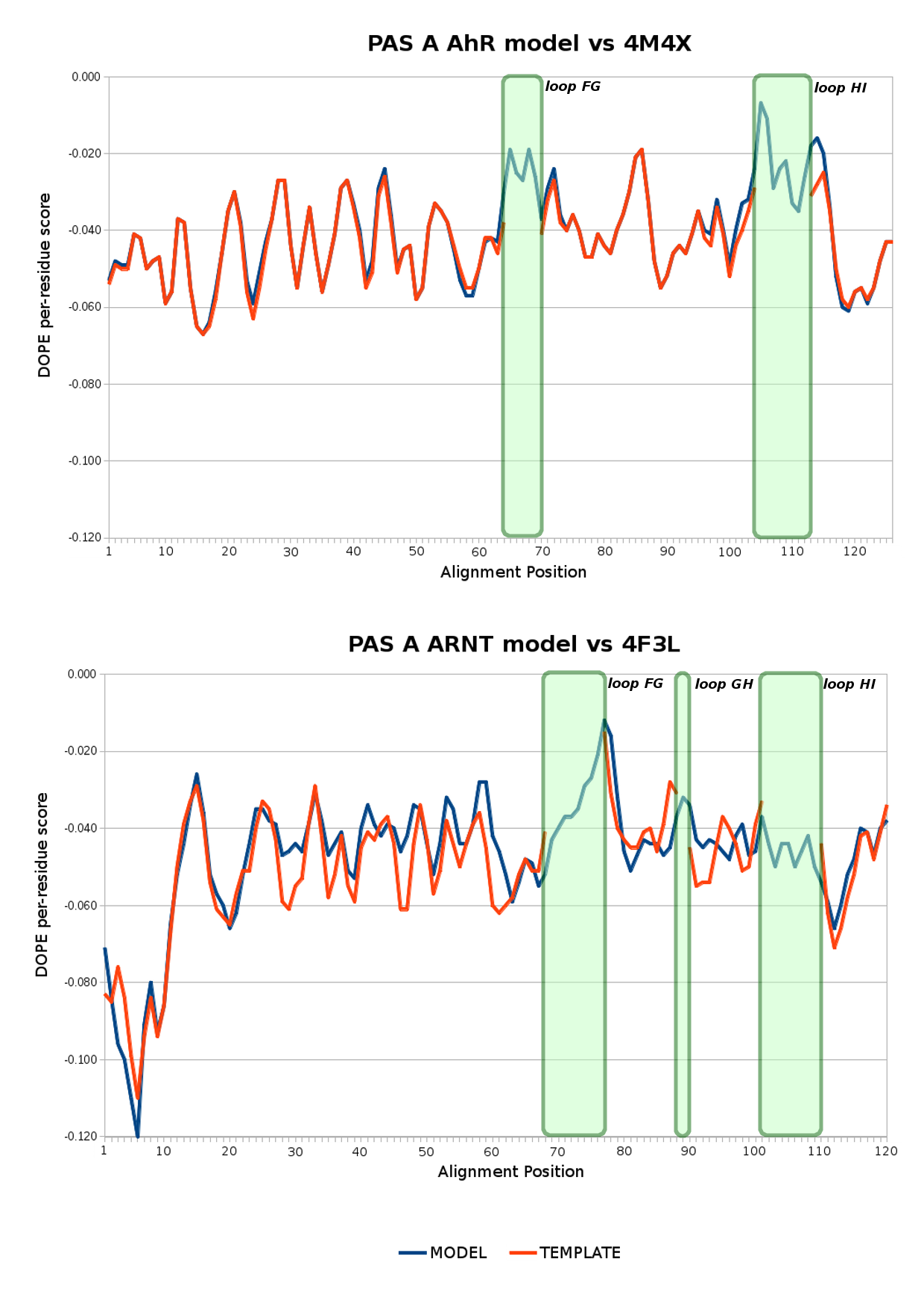

Supplement: S3 Fig — The protomer models of PAS-A domains (blue lines) are compared with the corresponding templates adopted (red lines). The green boxes highlight those regions that are not experimentally resolved in the structure of the template. (TIF) [file pcbi.1004981.s003.tif]

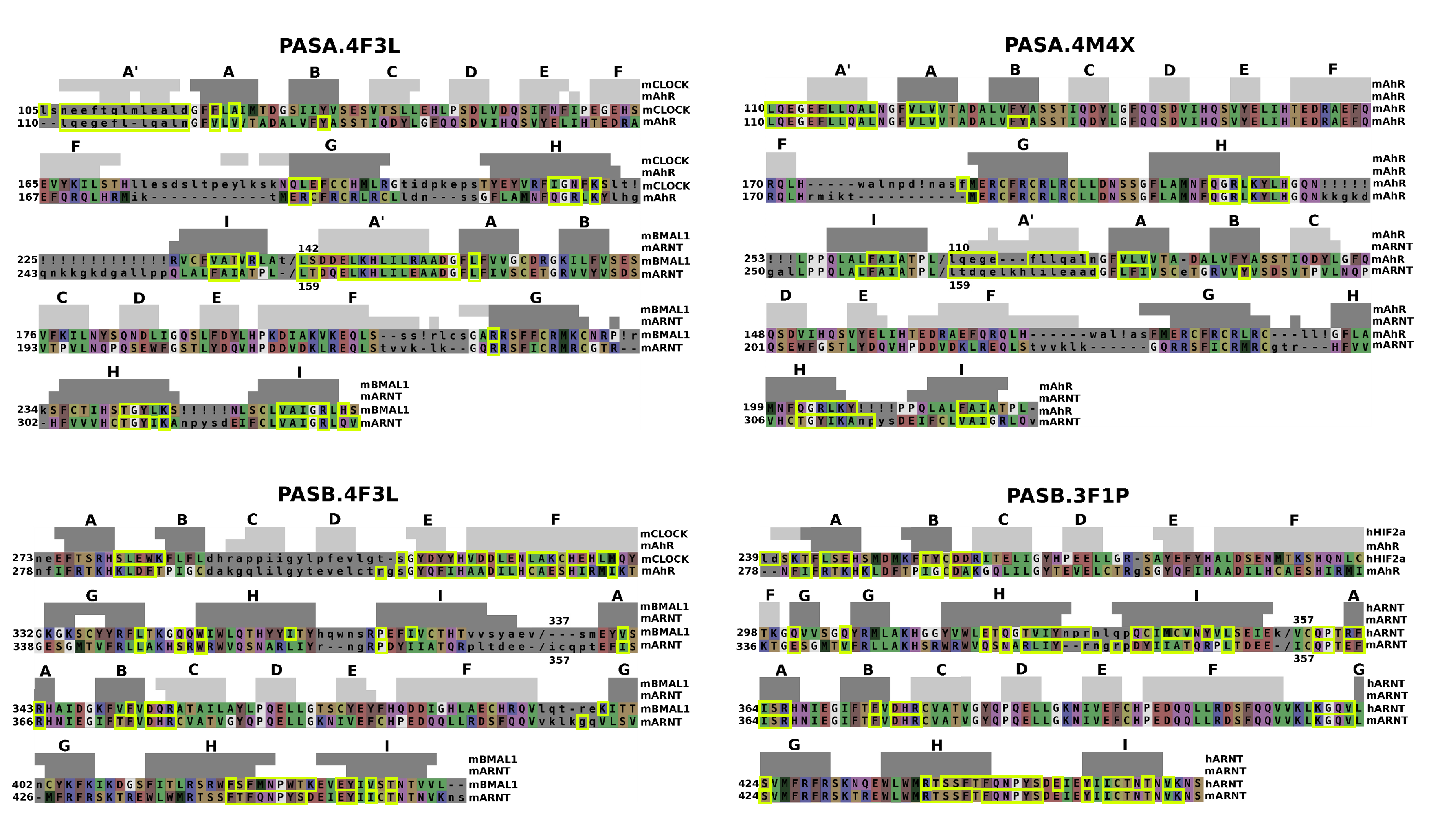

Supplement: S4 Fig — The lower case gray shaded residues highlight the regions that needed a refinement during modeling. The heading bars depict the secondary structure assignment according to PSIPRED prediction for the target sequence (mAhR/ARNT) or DSSPcont attribution over the structure of the templates. The light grey bars highlight the α-helices, the dark grey bars highlight the β-strands. Residues belonging to the PPI interfaces of both the models and the templates, as predicted by PISA, are highlighted by yellow boxes. (TIF) [file pcbi.1004981.s004.tif]

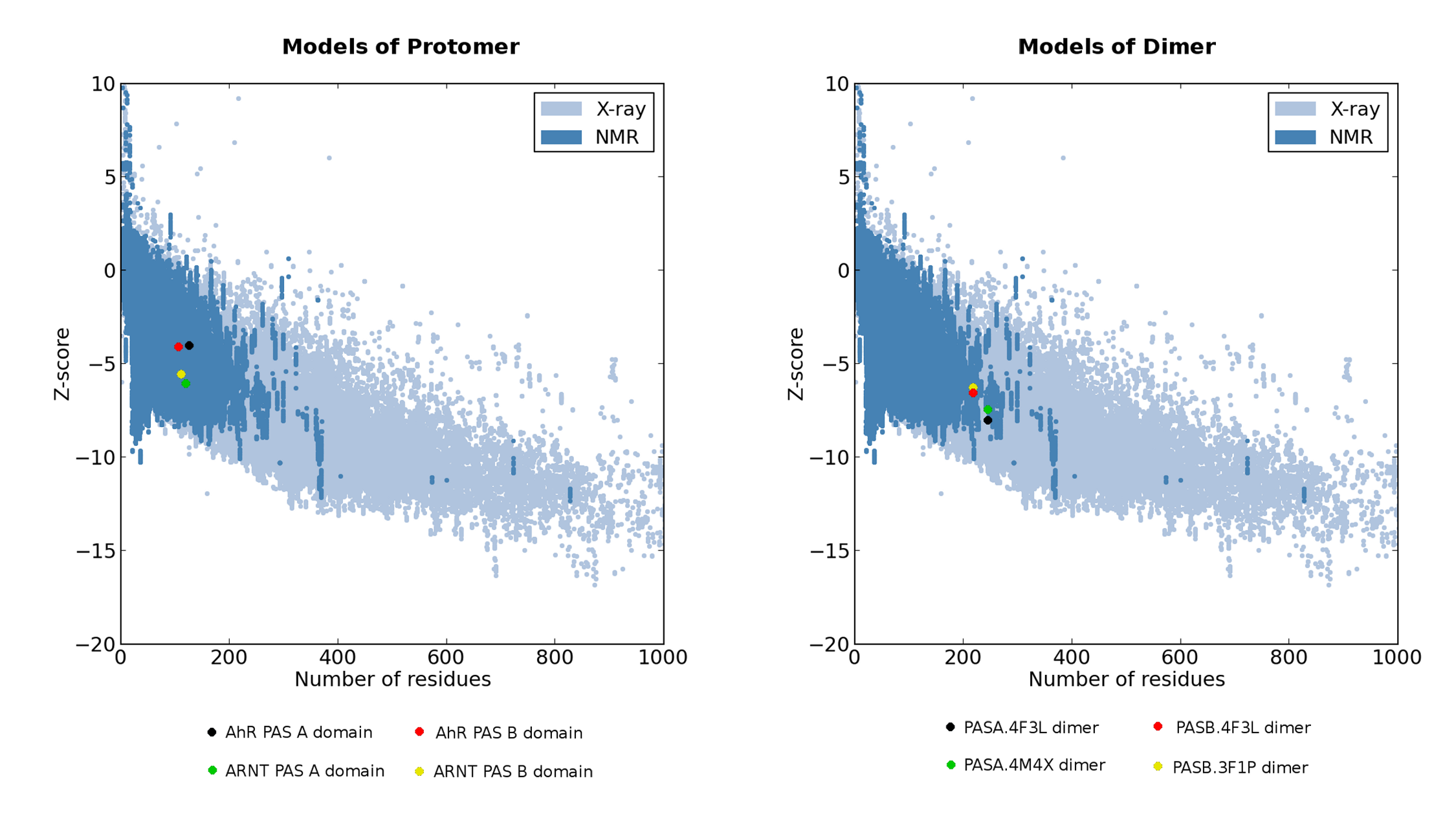

Supplement: S5 Fig — The distribution is represented along the sequence lengths taken from a dataset of experimentally resolved 3D protein structures. All of the models presented in this work (represented as black, yellow, green and red dots) fall into such distribution. (TIF) [file pcbi.1004981.s005.tif]

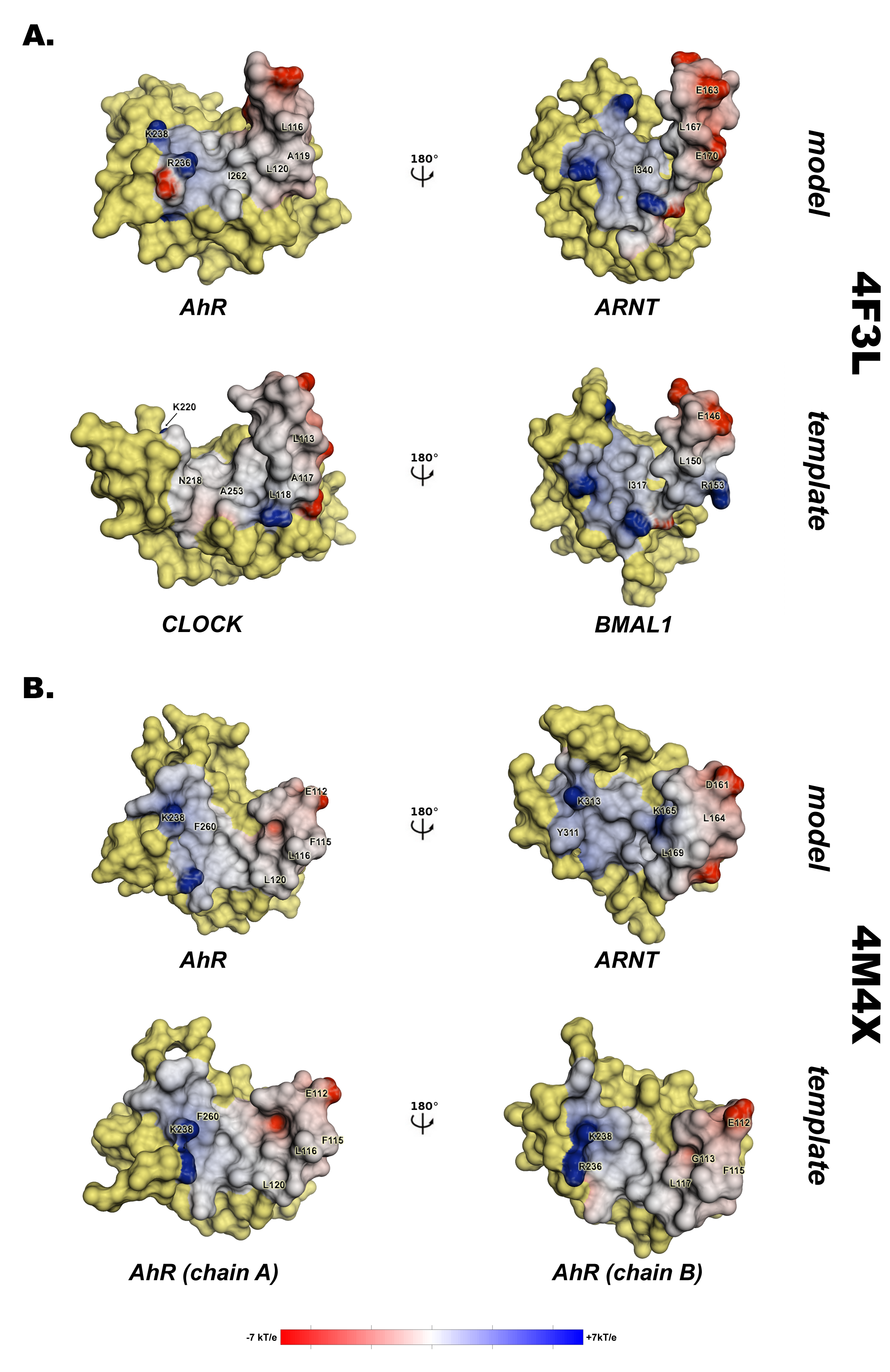

Supplement: S6 Fig — In each panel, the individual domains constituting the dimers are rotated each other of 180 degree, to obtain a representation as an “open book”. The potential range is defined in kT/e units according to the DelPhi software. Only the region defining the dimerization interface is colored according to the potential scale. The main residue contacts in the models (Fig 6) as well as the topological equivalent positions in the templates are labeled. (A) PASA.4F3L dimer model and CLOCK:BMAL1 template. (B) PASA.4M4X dimer model and AhR:AhR template. (TIF) [file pcbi.1004981.s006.tif]

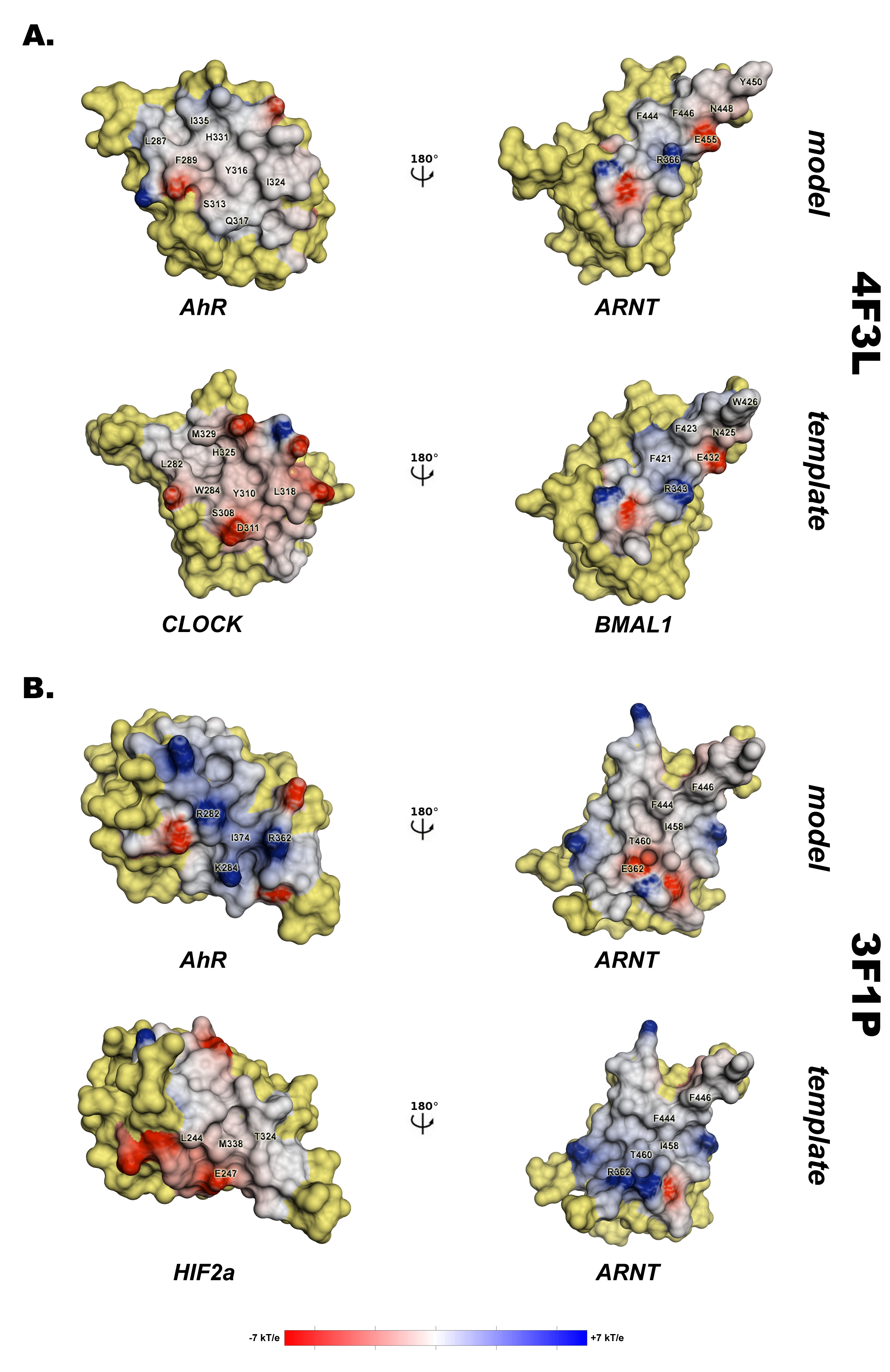

Supplement: S7 Fig — In each panel, the individual domains constituting the dimers are rotated each other of 180 degree, to obtain a representation as an “open book”. The potential range is defined in kT/e units according to the DelPhi software. Only the region defining the dimerization interface is colored according to the potential scale. The main residue contacts in the models (Fig 6) as well as the topological equivalent positions in the templates are labeled. (A) PASB.4F3L dimer model and CLOCK:BMAL1 template. (B) PASB.3F1P dimer model and HIF2α:ARNT template. (TIF) [file pcbi.1004981.s007.tif]

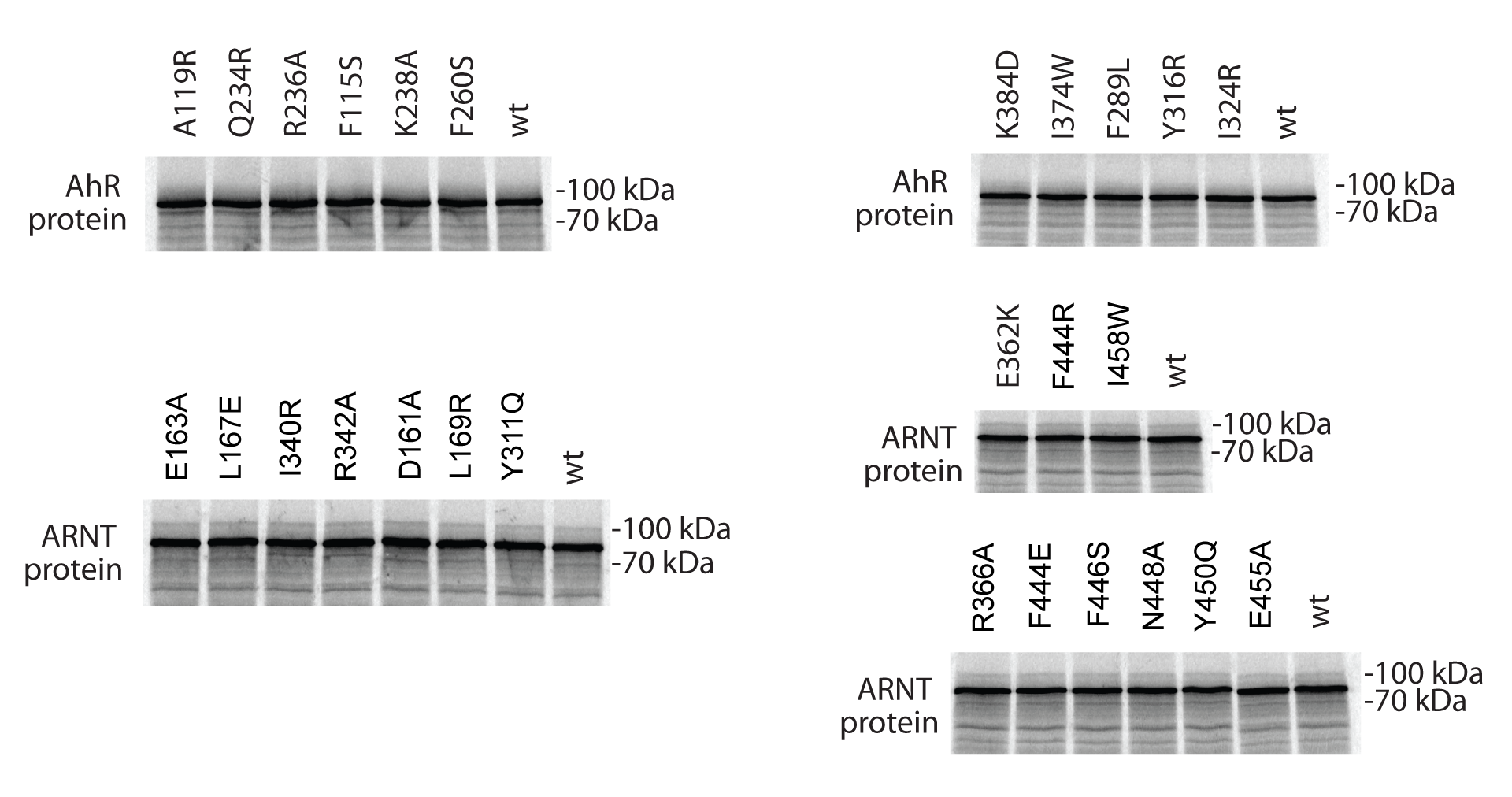

Supplement: S8 Fig — Indicated mutant AhR proteins were expressed in vitro in the presence of [35S]-methionine and resolved on SDS-PAGE gel and visualized by FLA9000 PSL (phospho-stimulated luminescence) analysis. (TIF) [file pcbi.1004981.s008.tif]

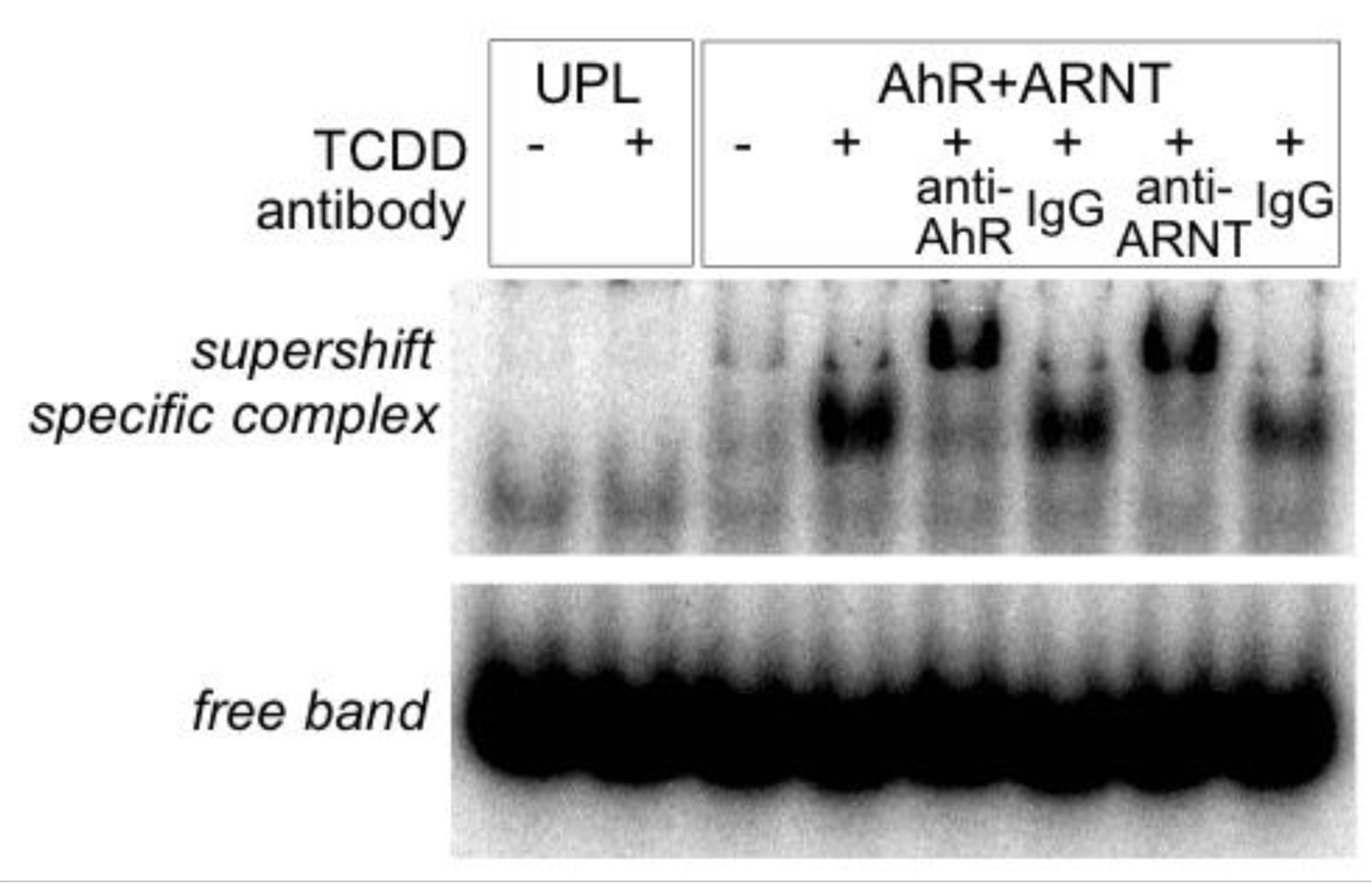

Supplement: S9 Fig — Mouse AhR and ARNT were separately synthesized in vitro using TNT lysate, transformed in the presence of 20 nM TCDD or solvent control DMSO (1% v/v) and analyzed for DNA binding with the EMSA assay. Where indicated, 400 ng of anti-AhR antibody (M20), anti-ARNT antibody (N19) or corresponding IgG control (all antibodies and controls from Santa Cruz Biotechnology) were added to the DNA-binding reaction. In the unprogrammed lysate (UPL) reactions, no plasmid DNA was included during the in vitro synthesis step resulting in the absence of AhR or ARNT protein. (TIF) [file pcbi.1004981.s009.tif]
