# Supplementary material for: Deciphering Dimerization Modes of PAS Domains: Computational and Experimental Analyses of the AhR:ARNT Complex Reveal New Insights Into the Mechanisms of AhR Transformation
Source: PLoS Comput Biol. 2016 Jun 13;12(6):e1004981. doi: 10.1371/journal.pcbi.1004981 (PMC4905635; doi:10.1371/journal.pcbi.1004981)
Supplement: S1 Table — (PDF) [file pcbi.1004981.s010.pdf]

Table S1: Sequence identity and similarity between the dimer models and the templates adopted.

Model	Identity (%)	Similarity (%)
PASA.4F3L	39.4 (57.7)	75.0 (67.3)
PASA.4M4X	65.0 (64.6)	75.0 (77.1)
PASB.4F3L	32.6 (43.5)	57.2 (67.4)
PASB.3F1P	64.2 (57.1)	75.7 (66.7)

The values reported in brackets are related to the aligned residues at the dimerization interfaces.
