# Supplementary material for: Deciphering Dimerization Modes of PAS Domains: Computational and Experimental Analyses of the AhR:ARNT Complex Reveal New Insights Into the Mechanisms of AhR Transformation
Source: PLoS Comput Biol. 2016 Jun 13;12(6):e1004981. doi: 10.1371/journal.pcbi.1004981 (PMC4905635; doi:10.1371/journal.pcbi.1004981)
Supplement: S2 Table — (PDF) [file pcbi.1004981.s011.pdf]

Table S2: Overall quality indices of the modeled structures.

Model	Type	RMSD[a] (Å)	Favored[b] (%)	G-factor[c]	Z-score[d]
PAS-A AhR	protomer	0.119	90.4	0.15	-4.03
PAS-A ARNT	protomer	0.859	92.5	-0.07	-6.07
PAS-B AhR	protomer	0.296	87.2	-0.12	-4.12
PAS-B ARNT	protomer	0.008	95.0	-0.06	-5.56
PASA.4F3L	dimer	1.060	94.1	0.01	-8.03
PASA.4M4X	dimer	0.783	93.6	-0.08	-7.45
PASB.4F3L	dimer	0.894	93.3	-0.18	-6.59
PASB.3F1P	dimer	0.338	93.3	-0.11	-6.28

[a] : values were computed on Cα atoms, by superposing the model against the template structure

[b] : amount of residues that fall in favored areas of the Ramachandran plot

[c] : score values from PROCHECK method

[d] : score values from ProSA method
