# Supplementary material for: Deciphering Dimerization Modes of PAS Domains: Computational and Experimental Analyses of the AhR:ARNT Complex Reveal New Insights Into the Mechanisms of AhR Transformation
Source: PLoS Comput Biol. 2016 Jun 13;12(6):e1004981. doi: 10.1371/journal.pcbi.1004981 (PMC4905635; doi:10.1371/journal.pcbi.1004981)
Supplement: S3 Table — (PDF) [file pcbi.1004981.s012.pdf]

Table S3: Rank Products profile for thePAS-A dimer models.

Residue	Domain	PPI[a]	LOG(*RP*)	*e-value*
K165	ARNT	both	-0.057	≤ 0.05
R236	AhR	both	-0.057	≤ 0.05
L167	ARNT	both	-0.024	≤ 0.05
L110	AhR	both	-0.023	≤ 0.05
Q234	AhR	both	-0.018	≤ 0.05
L159	ARNT	both	-0.018	≤ 0.05
A119	AhR	both	-0.015	≤ 0.05
L120	AhR	both	-0.014	≤ 0.05
I262	AhR	both	-0.013	≤ 0.05
A171	ARNT	both	-0.011	≤ 0.05
Y311	ARNT	both	-0.004	0.20
K238	AhR	both	-0.003	0.37
I168	ARNT	both	-0.002	0.38
I340	ARNT	both	-0.002	0.50
L117	AhR	both	-0.002	0.57
L116	AhR	both	-0.001	0.79
K313	ARNT	both	0.001	0.70
L164	ARNT	both	0.003	0.33
V338	ARNT	both	0.006	≤ 0.05
D161	ARNT	*PASA.4M4X*	0.008	≤ 0.05
L240	AhR	*PASA.4M4X*	0.009	≤ 0.05
L169	ARNT	both	0.009	≤ 0.05
F115	AhR	both	0.013	≤ 0.05
Y135	AhR	both	0.016	≤ 0.05
F260	AhR	both	0.018	≤ 0.05

[a] : this column defines at which dimerization interface (predicted by PISA) the specific residue belongs to
