# Supplementary material for: Deciphering Dimerization Modes of PAS Domains: Computational and Experimental Analyses of the AhR:ARNT Complex Reveal New Insights Into the Mechanisms of AhR Transformation
Source: PLoS Comput Biol. 2016 Jun 13;12(6):e1004981. doi: 10.1371/journal.pcbi.1004981 (PMC4905635; doi:10.1371/journal.pcbi.1004981)
Supplement: S4 Table — (PDF) [file pcbi.1004981.s013.pdf]

Table S4: *Hot spot* list from PAS-A dimer templates, with related scores from the PPI prediction tools herein adopted.

**CLOCK:BMAL1 (4F3L template)**				**AhR:AhR (4M4X template)**			
**Residue**	**Robetta**	**HotPoint**	**KFC2-A**	**Residue**	**Robetta**	**HotPoint**	**KFC2-A**
CLOCK:E109	*na*	*na*	1.14	AhR[A]:F115 (AhR:F115)	*na* (2.53)	*na* (17.12)	0.37 (-0.30)
CLOCK:G110	1.27	31.64	-0.07	AhR[A]:L116 **(AhR:L116)**	0.57 (2.41)	6.03 (28.67)	0.35 (0.71)
CLOCK:L113 **(AhR:L116)**	*na* (1.26)	*na* (26.53)	1.44 (0.73)	AhR[A]:L117 (AhR:L117)	0.36 (0.44)	34.42 (41.45)	-0.19 (0.80)
CLOCK:M114	0.59	21.61	0.76	AhR[A]:L120 **(AhR:L120)**	1.11 (1.40)	25.37 (33.14)	-0.68 (-0.01)
CLOCK:L115 (AhR:L117)	0.30 (0.68)	29.63 (39.46)	-1.57 (0.01)	AhR[A]:V124 (AhR:V124)	0.36 (0.53)	28.45 (38.39)	-0.81 (0.17)
CLOCK:A117 (AhR:A119)	*na* (*na*)	*na* (12.33)	0.90 (0.10)	AhR[A]:F115 **(AhR:F260)**	*na* (2.21)	*na* (27.04)	0.05 (1.20)
CLOCK:A118 **(AhR:L120)**	1.72 (1.97)	27.11 (39.34)	1.02 (1.35)	AhR[B]:E112 (ARNT:D161)	*na* (-0.05)	*na* (13.08)	0.84 (0.54)
CLOCK:F122	1.95	33.92	-0.10	AhR[B]:F115	*na*	*na*	0.40
CLOCK:Y184	1.91	17.60	0.06	AhR[B]:L116 (ARNT:I168)	0.56 (2.18)	6.03 (23.52)	0.44 (0.81)
CLOCK:I216	1.02	12.73	0.08	AhR[B]:L117 **(ARNT:L169)**	0.41 (0.73)	41.58 (38.99)	-0.49 (-0.01)
CLOCK:V252 (AhR:F260)	*na* (0.48)	*na* (25.43)	0.28 (0.03)	AhR[B]:L120	1.12	25.37	-0.86
CLOCK:T254 **(AhR:I262)**	*na* (0.95)	*na* (43.23)	1.00 (1.13)	AhR[B]:V124 (ARNT:L176)	0.37 (0.48)	28.45 (48.69)	-1.25 (-0.99)
BMAL1:E146 (ARNT:E163)	-0.27 (-0.45)	13.23 (11.28)	0.45 (0.74)				
BMAL1:L150 **(ARNT:L167)**	1.02 (1.89)	21.54 (38.70)	1.52 (1.13)				
BMAL1:I151 (ARNT:I168)	1.83 (0.81)	38.99 (39.90)	2.01 (0.54)				
BMAL1:R153	1.36	17.85	0.64				
BMAL1:A154 (ARNT:A171)	*na* (*na*)	9.47 (25.54)	1.64 (-0.07)				
BMAL1:G157	*na*	31.74	*na*				
BMAL1:V315	*na*	*na*	0.38				
BMAL1:I317 **(ARNT:I340)**	*na* (0.98)	*na* (30.43)	1.04 (0.42)				

na : residue not predicted as hot spot for the specific method

Residues and values in brackets identify topological equivalent residues of the dimer model also predicted as *hot spot*.

Residue highlighted in bold are predicted to mostly contribute to the dimer model stabilization (Table 2).
