# Supplementary material for: Deciphering Dimerization Modes of PAS Domains: Computational and Experimental Analyses of the AhR:ARNT Complex Reveal New Insights Into the Mechanisms of AhR Transformation
Source: PLoS Comput Biol. 2016 Jun 13;12(6):e1004981. doi: 10.1371/journal.pcbi.1004981 (PMC4905635; doi:10.1371/journal.pcbi.1004981)
Supplement: S5 Table — (PDF) [file pcbi.1004981.s014.pdf]

Table S5: Rank Products profile for the PAS-B dimer models.

**Residue**	**Domain**	**PPI**[a]	**LOG(*RP*)**	***e-value***
R282	AhR	*PASB.3F1P*	-0.075	≤ 0.05
K284	AhR	*PASB.3F1P*	-0.069	≤ 0.05
R362	AhR	*PASB.3F1P*	-0.055	≤ 0.05
R379	ARNT	both	-0.041	≤ 0.05
N448	ARNT	both	-0.038	≤ 0.05
R378	AhR	*PASB.3F1P*	-0.034	≤ 0.05
Y316	AhR	*PASB.4F3L*	-0.027	≤ 0.05
N278	AhR	none	-0.023	≤ 0.05
I324	AhR	*PASB.4F3L*	-0.021	≤ 0.05
K297	AhR	none	-0.021	≤ 0.05
P449	ARNT	both	-0.021	≤ 0.05
E339	AhR	*PASB.3F1P*	-0.017	≤ 0.05
L325	AhR	*PASB.4F3L*	-0.015	≤ 0.05
A321	AhR	*PASB.4F3L*	-0.011	≤ 0.05
I357	ARNT	none	-0.009	≤ 0.05
Y456	ARNT	both	-0.005	0.15
R366	ARNT	both	-0.002	0.60
I458	ARNT	both	0.003	0.45
I364	AhR	*PASB.3F1P*	0.012	≤ 0.05
L287	AhR	*PASB.4F3L*	0.013	≤ 0.05
P360	ARNT	*PASB.3F1P*	0.013	≤ 0.05
T376	AhR	*PASB.3F1P*	0.014	≤ 0.05
I374	AhR	*PASB.3F1P*	0.015	≤ 0.05
Y372	AhR	*PASB.3F1P*	0.015	≤ 0.05
E455	ARNT	*PASB.4F3L*	0.016	≤ 0.05
F375	ARNT	both	0.016	≤ 0.05
F446	ARNT	both	0.019	≤ 0.05
D377	ARNT	both	0.042	≤ 0.05
F444	ARNT	both	0.044	≤ 0.05
E362	ARNT	*PASB.3F1P*	0.051	≤ 0.05

[a] : this column defines at which dimerization interface (predicted by PISA) the specific residue belongs to
