# Supplementary material for: Deciphering Dimerization Modes of PAS Domains: Computational and Experimental Analyses of the AhR:ARNT Complex Reveal New Insights Into the Mechanisms of AhR Transformation
Source: PLoS Comput Biol. 2016 Jun 13;12(6):e1004981. doi: 10.1371/journal.pcbi.1004981 (PMC4905635; doi:10.1371/journal.pcbi.1004981)
Supplement: S6 Table — (PDF) [file pcbi.1004981.s015.pdf]

Table S6: *Hot spot* list from PAS-B dimer templates, with related scores from the PPI prediction tools herein adopted.

**CLOCK:BMAL1 (4F3L template)**				**HIF2a:ARNT (3F1P template)**			
**Residue**	**Robetta**	**HotPoint**	**KFC2-A**	**Residue**	**Robetta**	**HotPoint**	**KFC2-A**
CLOCK:W284	2.26	43.78	0.28	HIF2a:T243 (AhR:I280)	*na* (0.53)	*na* (28.79)	0.01 (-0.62)
CLOCK:Y310 **(AhR:Y316)**	3.38 (2.46)	33.54 (32.79)	1.36 (0.86)	HIF2a:L245 (AhR:R282)	1.43 (-0.56)	52.11 (32.56)	1.30 (1.47)
CLOCK:V315	0.94	17.92	0.33	HIF2a:E247 (AhR:K284)	6.02 (-1.02)	29.52 (19.33)	0.89 (1.73)
CLOCK:L318 **(AhR:I324)**	1.57 (1.40)	36.34 (34.42)	0.73 (0.55)	HIF2a:Y256	1.73	22.29	-0.10
CLOCK:H325 (AhR:H331)	2.95 (1.99)	26.99 (30.18)	0.23 (-0.48)	HIF2a:D258	2.59	11.20	-0.58
CLOCK:L328	0.32	32.99	-0.77	HIF2a:R260	2.64	19.85	-1.38
CLOCK:I373	0.57	38.91	-0.31	HIF2a:Q301	2.82	20.35	-1.28
BMAL1:V341	0.32	27.66	-1.36	HIF2a:Q322	1.23	30.74	-0.62
BMAL1:F421 (ARNT:F444)	1.06 (1.15)	20.01 (*na*)	-1.03 (-1.28)	HIF2a:T324 (AhR:R362)	2.17 (-0.47)	14.44 (21.76)	0.30 (1.50)
BMAL1:F423 **(ARNT:F446)**	2.96 (1.75)	21.74 (31.41)	0.95 (0.70)	HIF2a:I326 (AhR:I364)	1.22 (0.37)	29.40 (21.02)	0.71 (0.90)
BMAL1:N425 (ARNT:N448)	1.46 (0.80)	7.70 (17.14)	0.43 (0.00)	HIF2a:C336	-0.11	26.79	-0.19
BMAL1:W427 **(ARNT:Y450)**	6.93 (1.60)	39.53 (20.23)	0.98 (0.40)	HIF2a:M338 **(AhR:I374)**	0.61 (1.12)	38.35 (33.85)	1.43 (0.71)
BMAL1:Y433	1.34	26.92	0.31	HIF2a:V340 (AhR:T376)	1.01 (0.12)	33.70 (21.10)	1.34 (0.81)
BMAL1:V435 (ARNT:I458)	0.52 (0.57)	24.78 (26.09)	0.72 (-0.26)	HIF2a:Y342	1.77	30.79	-0.31
				ARNT:R362 (ARNT:E362)	0.74 (0.21)	21.43 (25.51)	0.28 (0.25)
				ARNT:I364 (ARNT:I364)	1.47 (0.07)	36.39 (37.43)	1.21 (1.07)
				ARNT:S365	*na*	*na*	0.09
				ARNT:R366	0.18	24.17	-0.34
				ARNT:D377	2.60	12.96	-0.79
				ARNT:R379	8.29	19.07	-0.91
				ARNT:S442 (ARNT:S442)	-0.06 (-0.35)	14.56 (15.98)	0.02 (0.49)
				ARNT:F444 **(ARNT:F444)**	2.11 (0.63)	45.94 (30.42)	0.86 (0.63)
				ARNT:F446 **(ARNT:F446)**	2.21 (1.57)	30.84 (28.20)	1.13 (0.66)
				ARNT:N448	1.32	9.13	-0.66
				ARNT:I458 (ARNT:I458)	1.02 (-0.67)	34.22 (33.85)	1.38 (1.40)
				ARNT:T460 (ARNT:T460)	0.13 (-0.06)	*na* (25.72)	1.08 (1.43)

na : residue not predicted as hot spot for the specific method

Residues and values in brackets identify topological equivalent residues of the dimer model also predicted as *hot spot*.

Residue highlighted in bold are predicted to mostly contribute to the dimer model stabilization (Table 3).
