# Supplementary material for: Deciphering Dimerization Modes of PAS Domains: Computational and Experimental Analyses of the AhR:ARNT Complex Reveal New Insights Into the Mechanisms of AhR Transformation
Source: PLoS Comput Biol. 2016 Jun 13;12(6):e1004981. doi: 10.1371/journal.pcbi.1004981 (PMC4905635; doi:10.1371/journal.pcbi.1004981)
Supplement: S1 Appendix — (PDF) [file pcbi.1004981.s016.pdf]

Rank Products profiles from individual residue free energy contribution

The theoretical approach of positional methods can be applied to combine ranked lists in various biological problems, where general feature selection is required [1,2]. In the specific field of the computational chemistry, the Rank Porducts method [3] was successfully used in order to extract energetic deteminants from the 3D structure of protein-protein complexes [4,5]. In this paper, this method was adopted to classify the list of residues that contribute to the definition of the $\Delta G_{binding}$ of the dimer models here proposed.

Let us consider a data set composed of independent replicates for estimating the individual contribution (from a given residue g) to the overall $\Delta G_{binding}$ of a protein dimer. For each residue g found in K replicates we calculate the ranking position $r_{i,g}$ of residue g among the N_i residues of the ith replicate, sorted by their respective values. The sorted values are extracted from MM-GBSA per-residue energy decomposition analysis. In our specific case of study the data set of independent replicates is composed by the 100 putative dimer models produced from the homology modeling protocol and the list of the N_i residues is defined by only those residues that are found to provide a contribution greater or equal to ±1 kcal mol^{-1} in at least the 75 % of the replicates of the data set.

The Rank Porducts score R_g is calculated as follows:

$$R_g = \prod_{i=1}^{K} \frac{r_{i,g}}{N_i}$$

The R_g value is related to the combined probability of observing residue g ranked so high just by chance (e.g.: ″the third residue with the greatest contribution or better″). The condition under control here is the presence of a well defined binding mode (shared by all of the putative models considered), determining two states to be compared that are the alternative PPI interfaces deriving from the two kind of PAS dimer models proposed. Afterwards, the calculation of the R_g values was

applied for ranking residues in both models and a combined score (RP_g) was calculated through the two sample Rank Porducts variant [6]:

$$\log(RP_g) = \log(R_g^A) - \log(R_g^B)$$

The residues with the smallest RP_g values (negative $\log(RP_g)$ values) have a more stabilizing effect for the dimer model *B* than the dimer model *A*; viceversa, residues with the biggest RP_g values (positive $\log(RP_g)$ values) have a more stabilizing effect for the dimer model *A* than the dimer model *B*. Statistical significance of RP_g values have been assessed for multiple testing through 10,000 random permutations. In this work a RP_g value will be considered significant if it shows an *e-value* ≤ 0.05.

Energy (eigen)Decomposition analysis

The Energy Decomposition method allows to analyze and detect the residue couplings important for the stabilization of a structure [7]. The method provides a simplified view of residue-residue pair interactions, extracting the major contributions to the energetic stability of the input structure.

For a protein of *N* residues, the *NxN* matrix (termed *interaction energy matrix*) between pairs of residues can be built. Usually, the matrix so obtained is rather noisy and difficult to explain, then it is simplified by means of the eigenvalue decomposition. Analysis of the components of the eigenvector associated with the lowest eigenvalue was shown to identify residues behaving as strong interaction centers, characterized by components with an intensity higher than the threshold value $t = (1/N)^{1/2}$, corresponding to a *flat* normalized vector whose residues would all provide the same contribution [8]. The original *interaction energy matrix* is diagonalized and re-expressed in the form:

$$A_{ij} \cong \sum_{k=1}^{K} \lambda_k \, w_i^k \, w_j^k$$

where K is the number of selected eigenvectors; λ_k is the kth eigenvalue; w^k_i and w^k_j are the ith and the jth components of the associated normalized eigenvector. The list of eigenvectors is sorted according to their respective eigenvalues (increasing order), so λ_1 shows the most negative value associated to the highest contribution to stabilization. It was observed that small or single-domain proteins can be accurately described considering only the eigenvector associated to the first eigenvalue λ_1 [8]. This approximation is not suitable for more complicated cases, such as multi-domain structures and/or complexes like dimers. Indeed, it was observed that more eigenvectors are generally needed to completely represent all the interactions that are fundamental for the system stability [4,9].

In this work, to yeld a global and compact description of the energetic response upon dimerization the number of eigenvectors was selected so that at least the 75% of the cumulated variance of the original *interaction energy matrix* is explained.

In the previous applications, the Energy Decomposition method was mainly oriented to analyze the non-bonded energy network as a whole [5,10–13]. The adoption of the overall stabilization energy for building the *interaction energy matrix* was aimed to emphasize the major non-bonded interactions among those secondary structure elements that compose the core of the domain. On the other hand, in this way the “weak” interactions, such as those involved in the dimerization, could be hidden.

Since this work is mainly oriented to characterize the energetic determinants involved in the dimerization binding modes, the interaction energy matrix adopted herein is based on the decomposition of the ΔGbinding, calculated by means of the MM-GBSA method (see text).

References

1. Jurman G, Merler S, Barla A, Paoli S, Galea A, Furlanello C. Algebraic stability indicators for ranked lists in molecular profiling. Bioinformatics. 2008;24: 258–64. doi:10.1093/bioinformatics/btm550

2. Monwar MM, Gavrilova ML. Multimodal biometric system using rank-level fusion approach. IEEE Trans Syst Man Cybern B Cybern. 2009;39: 867–78. doi:10.1109/TSMCB.2008.2009071

3. Breitling R, Armengaud P, Amtmann A, Herzyk P. Rank products: a simple, yet powerful, new method to detect differentially regulated genes in replicated microarray experiments. FEBS Lett. 2004;573: 83–92. doi:10.1016/j.febslet.2004.07.055

4. Corrada D, Colombo G. Energetic and dynamic aspects of the affinity maturation process: characterizing improved variants from the bevacizumab antibody with molecular simulations. J Chem Inf Model. 2013;53: 2937–50. doi:10.1021/ci400416e

5. Corrada D, Morra G, Colombo G. Investigating allostery in molecular recognition: insights from a computational study of multiple antibody-antigen complexes. J Phys Chem B. 2013;117: 535–52. doi:10.1021/jp310753z

6. Koziol JA. The rank product method with two samples. FEBS Lett. 2010;584: 4481–4484. doi:10.1016/j.febslet.2010.10.012

7. Tiana G, Simona F, De Mori GMS, Broglia RA, Colombo G. Understanding the determinants of stability and folding of small globular proteins from their energetics. Protein Sci. 2004;13: 113–24. doi:10.1110/ps.03223804

8. Colacino S, Tiana G, Colombo G. Similar folds with different stabilization mechanisms: the cases of Prion and Doppel proteins. BMC Struct Biol. 2006;6: 17. doi:10.1186/1472-6807-6-17

9. Genoni A, Morra G, Colombo G. Identification of Domains in Protein Structures from the Analysis of Intramolecular Interactions. J Phys Chem B. 2012;116: 3331–43. doi:10.1021/jp210568a

10. Genoni A, Morra G, Merz KM, Colombo G. Computational study of the resistance shown by the subtype B/HIV-1 protease to currently known inhibitors. Biochemistry. 2010;49: 4283–95. doi:10.1021/bi100569u

11. Morra G, Colombo G. Relationship between energy distribution and fold stability: Insights from molecular dynamics simulations of native and mutant proteins. Proteins. 2008;72: 660–72. doi:10.1002/prot.21963

12. Ragona L, Colombo G, Catalano M, Molinari H. Determinants of protein stability and folding: comparative analysis of beta-lactoglobulins and liver basic fatty acid binding protein. Proteins. 2005;61: 366–76. doi:10.1002/prot.20493

13. Scarabelli G, Morra G, Colombo G. Predicting interaction sites from the energetics of isolated proteins: a new approach to epitope mapping. Biophys J. 2010;98: 1966–75. doi:10.1016/j.bpj.2010.01.014
